# Effects of Brussels Chicory and Concurrent Exercise Training on HDL Function and Subclass Profiles in Overweight College Students: A Pilot Randomized Controlled Trial

**DOI:** 10.3390/nu18162678

**Published:** 2026-08-16

**Authors:** Ping Qu, Ting Zhu, Guanyu Chen, Yu Zhang, Yihan Wang, Qiuhui Xu, Yang Liu, Dongliang Wang

**Affiliations:** 1 Department of Sports, Sun Yat-sen University, Guangzhou 510275, China; quping@mail.sysu.edu.cn (P.Q.); zhut65@mail.sysu.edu.cn (T.Z.); zhangy976@alumni.sysu.edu.cn (Y.Z.); liuy758@alumni.sysu.edu.cn (Y.L.); 2 Department of Nutrition, School of Public Health, Sun Yat-sen University, Northern Campus, Guangzhou 510080, China; chengy268@alumni.sysu.edu.cn (G.C.); wangyh266@mail2.sysu.edu.cn (Y.W.); xuqh36@mail2.sysu.edu.cn (Q.X.)

**Keywords:** high-density lipoprotein, cholesterol efflux capacity, high-density lipoprotein subclass profile, concurrent exercise training, mindfulness

## Abstract

**Background**: To evaluate the effects of three lifestyle modification strategies—dietary supplementation with phenolic acid-rich Brussels chicory (Cho), Brussels chicory supplementation combined with supervised concurrent resistance exercise and high-intensity interval training (Cho-CT) or mindfulness-enhanced concurrent resistance exercise and high-intensity interval training (Cho-MeCT)—on high-density lipoprotein cholesterol efflux capacity (HDL-CEC) from macrophages and HDL subclass profiles (pre-β-HDL, HDL_3_, HDL_2_) in overweight adults. **Methods**: This study is an 8-week randomized controlled trial in which 97 overweight college students were randomized to Cho, Cho-CT, Cho-MeCT or control group. HDL-CEC from macrophages, the primary outcome, HDL subclass profiles, plasma activities of lecithin, such as cholesterol acyltransferase (LCAT) and cholesteryl ester transfer protein (CETP) involved in remodeling HDL subclass profiles and plasma lipid profiles, and apolipoprotein AI were assessed at baseline and post-intervention. **Results**: Compared with the control treatment, all three lifestyle interventions comparably elicited a 1.18% to 1.78% elevations in HDL-CEC from macrophages (*p* < 0.001). Their improvements were accompanied by reductions in pre-β-HDL and HDL_3_ concentrations and increased HDL_2_ concentrations. However, there were no significant differences in plasma lipid profiles, apolipoprotein A-I, and plasma LCAT and CETP activities among the tested groups. Within the Cho, Cho-CT, and Cho-MeCT groups, changes in HDL-CEC from macrophages were positively correlated with changes in HDL_2_ levels, whereas weak negative correlations were observed for pre-β-HDL and HDL_3_, though these associations did not reach statistical significance. No comparable significant correlations were found in the control group. **Conclusions**: In overweight adults, sustained Brussels chicory supplementation, either alone or combined with concurrent exercise training, may yield comparable favorable changes in HDL-CEC from macrophages and HDL subclass profiles, though effect magnitudes appear modest in this pilot trial.

## 1. Introduction

Overweight and obesity have become a global public-health epidemic and have been increasingly recognized as independent risk factors for atherosclerotic cardiovascular disease (ASCVD), the leading cause of morbidity and mortality worldwide [1]. Epidemiological studies have strongly suggested that the proatherogenic effects of overweight and obesity stem in part from dysregulated lipid metabolism, including reduced circulating high-density lipoprotein (HDL) levels [2]. Beyond lower HDL cholesterol concentrations, overweight and obesity also reduce HDL cholesterol efflux capacity (HDL-CEC) from macrophages, the major cell types loaded with cholesterol in atherosclerotic plaques; this functional deficit often coincides with shifts in HDL subclass distribution, namely elevated pre-β-HDL and HDL_3_ concentrations, and decreased HDL_2_ levels [3,4,5,6]. Accordingly, counteracting overweight- and obesity-related quantitative and qualitative HDL dysfunction holds promise for slowing the onset and progression of ASCVD.

Healthy dietary patterns and regular physical activity are core lifestyle strategies to mitigate overweight and obesity and metabolic complications, including HDL deficits [7,8,9]. Traditional dietary approaches rely heavily on calorie restriction to prevent overweight and obesity and associated HDL dysfunction, yet poor adherence limits their utility [10]. Functional foods have emerged as an appealing alternative, capable of improving HDL quantity and function independent of calorie restriction [11]. In terms of physical activity, concurrent resistance exercise and high-intensity interval training (HIIT) has garnered substantial research interest, as it favorably remodels body composition and improves HDL function [12,13,14,15]. Sanllorente et al. reported that a 6-month intervention with an energy-restricted Mediterranean diet plus physical activity improved HDL functionality on the triglyceride metabolism in older adults with metabolic syndrome compared with a nonrestrictive Mediterranean diet without physical activity [16]. However, they did not evaluate the effect of an energy-restricted Mediterranean diet alone or physical activity alone on HDL functionality. While the separate lipid-modulating benefits of functional food supplementation and concurrent exercise training are well characterized, few studies have tested optimized combinations to amplify improvements in HDL function and subclass profiles, leaving effective diet–exercise synergies poorly defined.

Only a few dietary factors, such as saturated fats, alcohol, and, to a lesser extent, dietary cholesterol, are known to strongly increase HDL cholesterol, yet high intake of these components is not recommended for ASCVD prevention [17]. Beyond such drivers of HDL cholesterol levels, evaluating the effects of foods and bioactive compounds on HDL function is critical, even when HDL cholesterol concentrations remain unchanged, as these effects may confer atheroprotection [11,18]. Plant foods contain abundant bioactive non-nutrient polyphenolic compounds, which are hypothesized to underpin the inverse association between fruit and vegetable consumption and ASCVD risk [19,20]. Human observational and interventional trials have strongly suggested that several flavonoids (a major polyphenol class, particularly anthocyanins) appreciably increase HDL-CEC from macrophages [17,21]. By contrast, the impacts of phenolic acids, another key polyphenol subclass, on HDL quantity and function remain largely unstudied [22,23].

Brussels chicory (*Cichorium intybus* L. var. *foliosum*), also known as witloof chicory, Belgian endive, or French endive, is a typical leafy vegetable of the traditional Mediterranean diet [24]. Providing approximately 19.6 kcal per 100 g fresh weight, it is low-energy yet rich in multiple nutrients, including dietary fiber (prebiotic inulin), vitamin K, β-carotene, folate, and manganese [25]. Beyond these macro- and micro-nutrients, this vegetable displays abundant bioactive polyphenolic components, predominantly phenolic acids represented by protocatechuic acid, gallic acid, caffeic acid, 5-caffeoylquinic acid, caftaric acid, and chicoric acid [23]. Flavonoids and terpenoids are also detectable, albeit in smaller quantities. Our previous work in apolipoprotein E (ApoE)-deficient mice showed that long-term dietary supplementation of Brussels chicory attenuated atherosclerotic progression and lowered atherosclerotic plaque cholesterol burden; however, this vegetable did not change plasma lipid and lipoprotein profiles including HDL cholesterol [24,26,27]. This points to a potential mechanism whereby Brussels chicory facilitates cholesterol clearance from atherosclerotic plaques by boosting HDL-CEC from atherosclerotic plaque macrophages. Consistent with this hypothesis, our recent small-scale randomized controlled trial found that one week of dietary supplementation with Brussels chicory increased HDL-CEC from aortic endothelial cells in overweight adults, an effect likely secondary to favorable shifts in HDL subclasses with reduced pre-β-HDL and increased HDL_3_ [23].

To this end, we hypothesized that sustained Brussels chicory supplementation would increase HDL-CEC from macrophages and remodel HDL subclass profiles in overweight adults. To test this hypothesis, we recruited overweight college students to assess the effect of an eight-week dietary supplementation with Brussels chicory without dietary energy intake constraints on HDL-CEC from macrophages and HDL subclass profiles. Given that concurrent resistance exercise and HIIT also improve HDL-CEC from macrophages and HDL subclass profiles across diverse populations [28], we additionally evaluated the effects of combined interventions, namely Brussels chicory paired with supervised conventional concurrent exercise training and Brussels chicory paired with supervised mindfulness-enhanced concurrent exercise training, on HDL function and subclass profiles. Mindfulness was chosen because it was expected to improve participants’ adherence to the concurrent exercise training [29].

## 2. Materials and Methods

### 2.1. Study Design and Participants

This study was an 8-week, four-arm, randomized, controlled trial, which was approved by the Biomedical Research Ethics Review Committee of the School of Public Health, Sun Yat-sen University (No. 2024-088) and was registered at chictr.org.cn (No. ChiCTR2400082335). All procedures adhered to institutional guidelines and the Helsinki Declaration.

The inclusion criteria were as follows: (1) male college students from Sun Yat-sen University; (2) aged 17–30 years; and (3) overweight conditions (body mass index, BMI ≥ 24.0 kg/m^2^). Exclusion criteria included (1) prior regular mindfulness practice or structured exercise training (more than three times per week of moderate-to-vigorous exercise, and each exercise session lasting more than 20 min); (2) smokers who have smoked continuously or cumulatively for six months or more; (3) consumption ≥ 175 g of ethanol per week; (4) current or impending pharmacological treatment; (5) contraindications to exercise testing (e.g., severe hypertension, cardiovascular disease, peripheral vascular disorders, respiratory conditions) as assessed by the Physical Activity Readiness Questionnaire; (6) inability to complete HRPF tests or questionnaires; (7) noncompliance with trial protocols; (8) refusal to provide written informed consent; and (9) intolerance or allergy to milk products. All participants were volunteers who were entitled to withdraw from the study at any time without explanation and signed a written informed consent form. Any reported adverse events were recorded and monitored.

**Figure 1:**
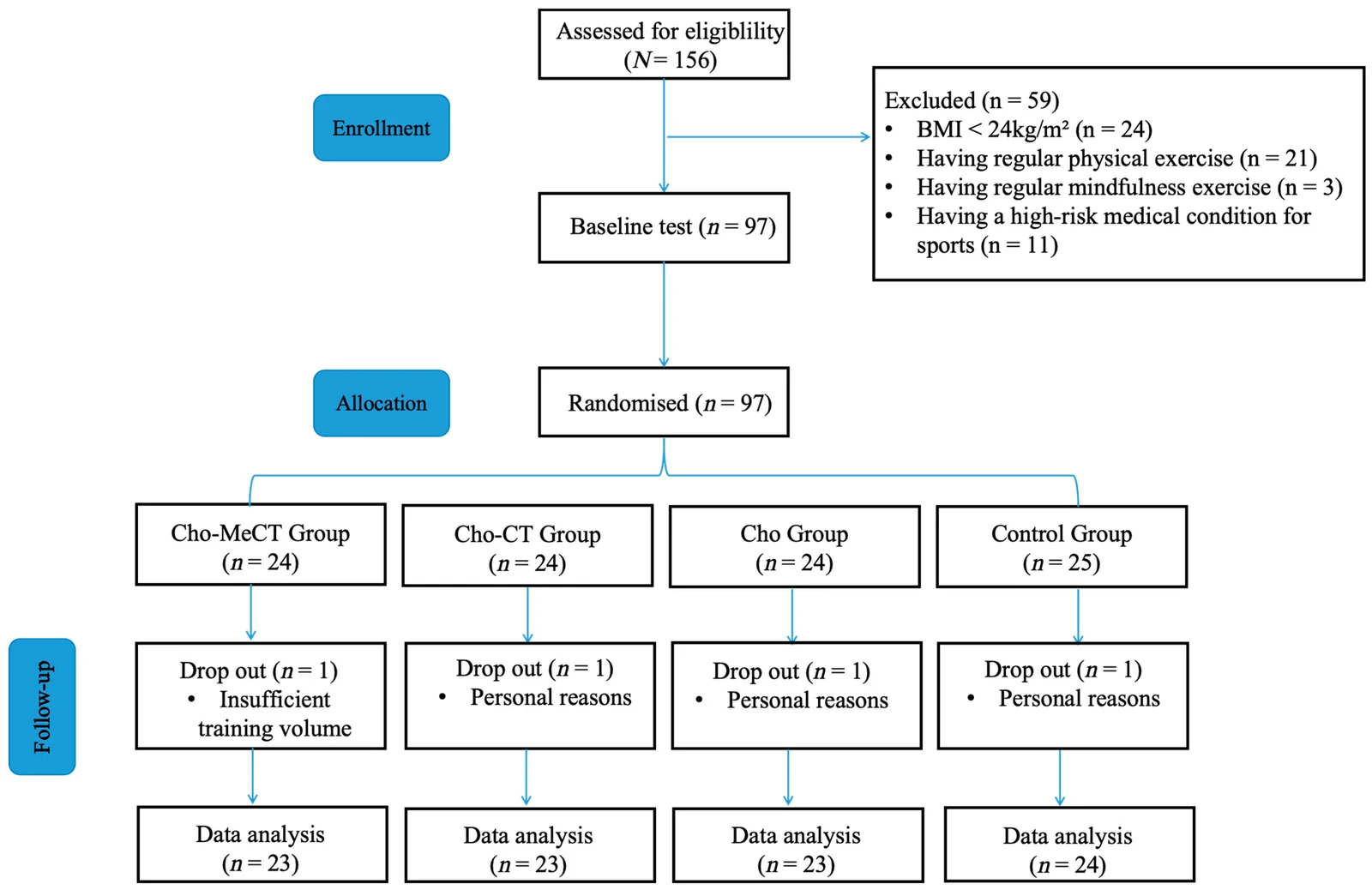
Participant screening, randomization, and follow-up.

The sample size required for each group was calculated according to the formula at an a level of 0.05 and a b level of 0.2, using a two-sided test. A total sample size of 100 participants was planned, including a 10% attrition rate. Of the first 156 undergraduate students screened, 97 were recruited and randomly assigned into the control (Con), Brussels chicory (Cho), Brussels chicory plus supervised concurrent exercise training integrating resistance exercise and high-intensity interval training (Cho-CT), or Brussels chicory plus supervised mindfulness-enhanced concurrent exercise training integrating resistance exercise and high-intensity interval training (Cho-MeCT). The sample size in the control group was set as 25, while the other groups displayed 24. Ninety-three participants completed the study, with one dropout per group (Figure 1).

### 2.2. Interventions

The participants in the Cho group were instructed to consume 100 mL of home-made Brussels chicory smoothie containing 50 g of fresh Brussels chicory (Hebei Lianxing Jiayao Agricultural Technology Co., Ltd., Shijiazhuang, China) and 15 g of whole milk powder (Inner Mongolia Mengniu Dairy (Group) Limited by Share Ltd., Hohhot, China) under supervision within 5 min after each lunch during the 8-week trial. The nutrient and polyphenolic composition profiles of Brussels chicory are detailed in Table S1, in which 50 g of fresh Brussels chicory contains 110.67 mg of phenolic acids (protocatechuic acid, 16.86 mg; gallic acid, 12.19 mg; caffeic acid, 4.66 mg; 5-caffeoylquinic acid, 49.13 mg; caftaric acid, 10.09 mg; chicoric acid, 17.74 mg). The participants in the Cho-CT group received co-treatment of Brussels chicory and supervised exercise. The exercise protocol was detailed in Tables S2 and S3. In brief, the concurrent training (CT) program consisted of high-intensity interval training and resistance training, performed three times per week for 90 min per session. The first 2 weeks served as an adaptation phase, followed by 6 weeks of intensification. High-intensity interval training was performed at 75–85% of maximal heart rate, with 8–20 exercises per session and 0–15 s rest intervals; resistance training used dumbbells, barbells, and elastic bands at 30–80% of one-repetition maximum (1RM), with 6–10 exercises, 8–20 repetitions per set, and 30–60 s inter-set rest intervals. The participants in the Cho-MeCT group received co-treatment of Brussels chicory and mindfulness-enhanced supervised concurrent exercise training, with the mindfulness components fully described in Tables S4 and S5. The participants in the control group consumed 100 mL home-made smoothie containing 15 g of whole milk powder. During the trial, all participants were instructed to maintain their usual diet and physical activity levels. To monitor adherence, dietary intake and physical activity outside of the exercise sessions were assessed at baseline and before the end of the intervention using a 3-day 24 h dietary recall and the Chinese version of the International Physical Activity Questionnaire, respectively [30]. To minimize dropout rates, participants in the control group were offered a delayed supervised exercise between 6 and 12 weeks after this trial. Moreover, participants who completed the intervention program received physical education course credits, which are required for all enrolled university students.

### 2.3. Blood Sampling

Blood samples collected from the elbow venous of the participants before and after the interventions in EDTA tubes were centrifuged at 8000× *g* for 5 min at 4 °C. Plasma samples were frozen in aliquots at −80 °C until analysis.

### 2.4. Primary Outcome

HDL-CEC from macrophages was determined by measuring the efflux of BODIPY-tagged cholesterol from J774A.1 murine macrophages (American Type Culture Collection, VA) to Apo B-depleted plasma as we previously described [31]. In brief, macrophages labeled with BODIPY-tagged cholesterol (Avanti Polar Lipids, Alabaster, AL, USA) in the presence of Sandoz 58-035 (acyl-coenzyme A: cholesterol acyltransferase inhibitor, Sigma-Aldrich, St. Louis, MO, USA) were incubated with 2.8% Apo B-depleted plasma in MEM-HEPES buffer, 0.3 mM cyclic adenosine monophosphate and 2 µg/mL Sandoz 58-035 for 4 h. The quantity of BODIPY cholesterol in the medium was determined with Infinite F200 (Tecan Group Ltd., Männedorf, Switzerland), and HDL-CEC from macrophages was calculated as the amount of effluxed BODIPY cholesterol expressed as a fraction of the initial cell content of BODIPY cholesterol. All samples were run in duplicate, and the average value was reported.

### 2.5. Secondary Outcomes

Body composition was measured using a body composition analyzer (InBody 570, InBody Co., Ltd., Seoul, South Korea) based on the bioelectrical impedance analysis method. Multi-frequency bioelectrical impedance analysis used by the InBody 570 scale is a valid method for determining body fat percentage when compared with dual-energy X-ray absorptiometry [32]. All measurements were performed by two well-trained examiners (blinded to the experimental groups) to minimize coefficients of variation.

Health-related physical fitness (HRPF) was measured as we previously described [33]. They include body fat percentage (body composition), push-ups (upper limb strength and endurance), curl-up (abdominal strength and endurance), sit-and-reach (flexibility), and endurance running (cardiorespiratory endurance). HRPF scores were calculated separately using composite scores based on China’s National Student Physical Health Standard (Ministry of Education of the People’s Republic of China, 2014). Specifically, an overall score for each aspect of physical health was calculated using Z-score and T-score conversions for each domain [34,35].

Mindfulness was assessed following the Chinese version of the 20-item Five Facet Mindfulness Questionnaire-Short Form as we previously described [36,37]. It was composed of the following five facets: observing, describing, acting with awareness, non-judging of inner experience, and non-reactivity to inner experience. Items were scored on a 5-point Likert scale ranging from 1 (never or very rarely true) to 5 (often or always true). Items 9–16 were reverse-coded. Scores were summed up, and higher scores indicated greater levels of mindfulness. The Cronbach’s α coefficients for the pre-test and post-test were 0.636 and 0.863, respectively.

The levels of plasma protocatechuic acid were determined by high-performance liquid chromatography with electrochemical detection as we previously described [38]. Plasma lipids and apolipoproteins [total cholesterol (TC), total triglyceride (TG), low-density lipoprotein cholesterol (LDL-C), HDL cholesterol (HDL-C)], alanine aminotransferase and aspartate aminotransferase, albumin, creatinine and urea nitrogen were routinely measured using a Cobas e602 automatic biochemical analyzer (311, Roche Diagnostics International AG, Rotkreuz, Switzerland). Plasma Apo A-I was measured by an enzyme-linked immunosorbent assay system using commercial kits (Elabscience Biotechnology Co., Ltd., Wuhan, China). Apo A-I contents of HDL subclasses (pre-β_1_-HDL, pre-β_2_-HDL, HDL_3c_, HDL_3b_, HDL_3a_, HDL_2a_, HDL_2b_) were determined by two-dimensional gel electrophoresis coupled with immunodetection for Apo A-I as described previously [39]. Plasma LCAT activity was assayed using a colorimetric method with autosubstrate in the presence of dipalmitoyl lecithin (Sigma Aldrich, St Louis, MO, USA). Plasma CETP activity was assayed by using fluorometric assay kit (Bio Vision Inc., Mountain View, CA, USA).

### 2.6. Statistical Analysis

Data entry was performed by two independent researchers using double entry and verification to ensure data quality. Data are presented as mean ± SD. All statistical analyses were conducted using Stata version 16.0 (StataCorp LP, College Station, TX, USA) and GraphPad Prism 10, with statistical significance set at a two-tailed *p* < 0.05.

Generalized estimating equations (GEE) were used to analyze repeated measures data at baseline, week 4, and week 8. For continuous outcomes, GEE models were fitted assuming a Gaussian distribution with an identity link function. GEE was selected because our primary interest was to estimate population-averaged intervention effects rather than subject-specific trajectories. Furthermore, GEE provides robust parameter estimates under potential misspecification of the working correlation structure. Covariates included baseline measurement values, time, group (Con, Cho, Cho-CT, Cho-MeCT), and the group-by-time interaction, which was considered the primary indicator of intervention effects. A first-order autoregressive (AR(1)) working correlation structure was specified to account for within-subject correlations across repeated measurements. This structure was selected based on the assumption that correlations between repeated measurements decrease as the interval between assessments increases. The appropriateness of the working correlation structure was evaluated using the quasi-likelihood under the independence model criterion (QIC), and AR(1) showed the lowest QIC value among the evaluated structures and was therefore selected for the final analyses. Pairwise comparisons between groups were performed using linear contrasts with Bonferroni correction. Effect sizes were expressed as mean differences (MD) with 95% confidence intervals (CI) for pairwise comparisons between groups.

Exploratory mediation analyses were conducted to assess whether the intervention-induced improvement in HDL-CEC from macrophages could be explained by concurrent changes in body weight (Δweight), or whether it occurred independently of weight loss. Each intervention group was compared with the control group. Bias-corrected bootstrapping with 5000 resamples was used to estimate indirect effects and their 95% confidence intervals; an indirect effect was considered statistically significant if the CI excluded zero.

To explore potential associations between intervention-induced changes in HDL-CEC from macrophages and related metabolic parameters, changes from baseline to week 8 in PCA, pre-β-HDL, HDL_3_, HDL_2_, and HDL-CEC from macrophages were calculated for each participant. Bivariate correlation analyses were performed using Spearman’s rank correlation coefficients. These analyses were considered exploratory and hypothesis-generating and were conducted as complementary analyses to the primary GEE models evaluating intervention effects, aiming to further characterize whether changes in HDL-CEC from macrophages were associated with concurrent alterations in related metabolic parameters. The correlation between changes in PCA and changes in HDL-CEC from macrophages was presented as a scatter plot. To account for multiple comparisons, Bonferroni corrections were applied separately for each set of correlations: an adjusted significance threshold of *p* = 0.0125 (0.05/4 comparisons) was applied for the correlation between PCA and HDL-CEC from macrophages, and *p* = 0.0042 (0.05/12 comparisons) was applied for the correlations between HDL-CEC from macrophages and HDL subclasses.

## 3. Results

### 3.1. Participant Characteristics and Intervention Fidelity

A total of 156 overweight male college students participated in the screening process (Figure 1). Of these, 59 did not meet the selection criteria, and 97 completed the baseline assessments. Between the baseline and post-intervention assessments, three participants withdrew from this study for personal reasons, and one participant in the Cho-MeCT group was excluded due to insufficient exercise volume (completing less than 85% of the set training volume each week) [40]. Ultimately, 93 participants were included in the final data analysis, with 23 in the Cho-MeCT group, 23 in the Cho-CT group, 23 in the Cho group, and 24 in the Con group.

As shown in Table 1, no significant differences were observed among the four groups (Con, Cho, Cho-CT, and Cho-MeCT) at baseline with respect to anthropometric measures (e.g., age and BMI), health-related physical fitness (HRPF and vital capacity), mindfulness levels, physical activities outside of exercise sessions, daily energy intake, or biochemical parameters (e.g., TC, HDL-C, and Apo A-I) (*p* > 0.05).

As shown in Table 2, there were increased levels of plasma protocatechuic acid (an indicator for Brussels chicory consumption), HRPF and vital capacity (two well-known indicators for concurrent exercise training), or mindfulness (an indicator for the mindfulness intervention) after Brussels chicory, concurrent exercise training or mindfulness intervention, respectively, whereas these levels in the Con group were similar during the 8-week trial period. During the 8-week intervention period, no abdominal pain, bloating, vomiting, headache, or diarrhea, were reported by any participant. Moreover, no differences in physical activities outside of exercise sessions and daily energy intake and also in liver and kidney function among the tested groups were observed during the 8-week trial period (Table S6).

Analysis of anthropometric measures further revealed that, compared with the Con and Cho groups, both the Cho-MeCT and Cho-CT groups exhibited significant reductions in body weight at week 4 (Tables S6 and S7). By week 8, however, only the Cho-MeCT group remained significantly lower than the two non-exercise groups (Tables S6 and S7). Regarding body fat percentage, the Cho-MeCT group showed significantly lower values than the Con and Cho groups at both week 4 and week 8, whereas the Cho-CT group was significantly lower than the Con and Cho groups only at week 8 (Tables S6 and S7). For waist circumference, the Cho-MeCT group was significantly lower than the Con and Cho groups at both week 4 and week 8; although the Cho-CT group did not reach statistical significance, it exhibited a sustained decreasing trend and approached marginal significance at week 8 (*p* = 0.056–0.058; Tables S6 and S7). In terms of hip circumference, the Cho-MeCT group was significantly lower than the Con group at both week 4 and week 8 and was also significantly lower than the Cho group at week 8; the Cho-CT group was significantly lower than both the Con and Cho groups at week 8 (Tables S6 and S7). Collectively, these findings suggest that the compliance of the participants with this trial was very good.

### 3.2. Primary Outcomes

**Figure 2:**
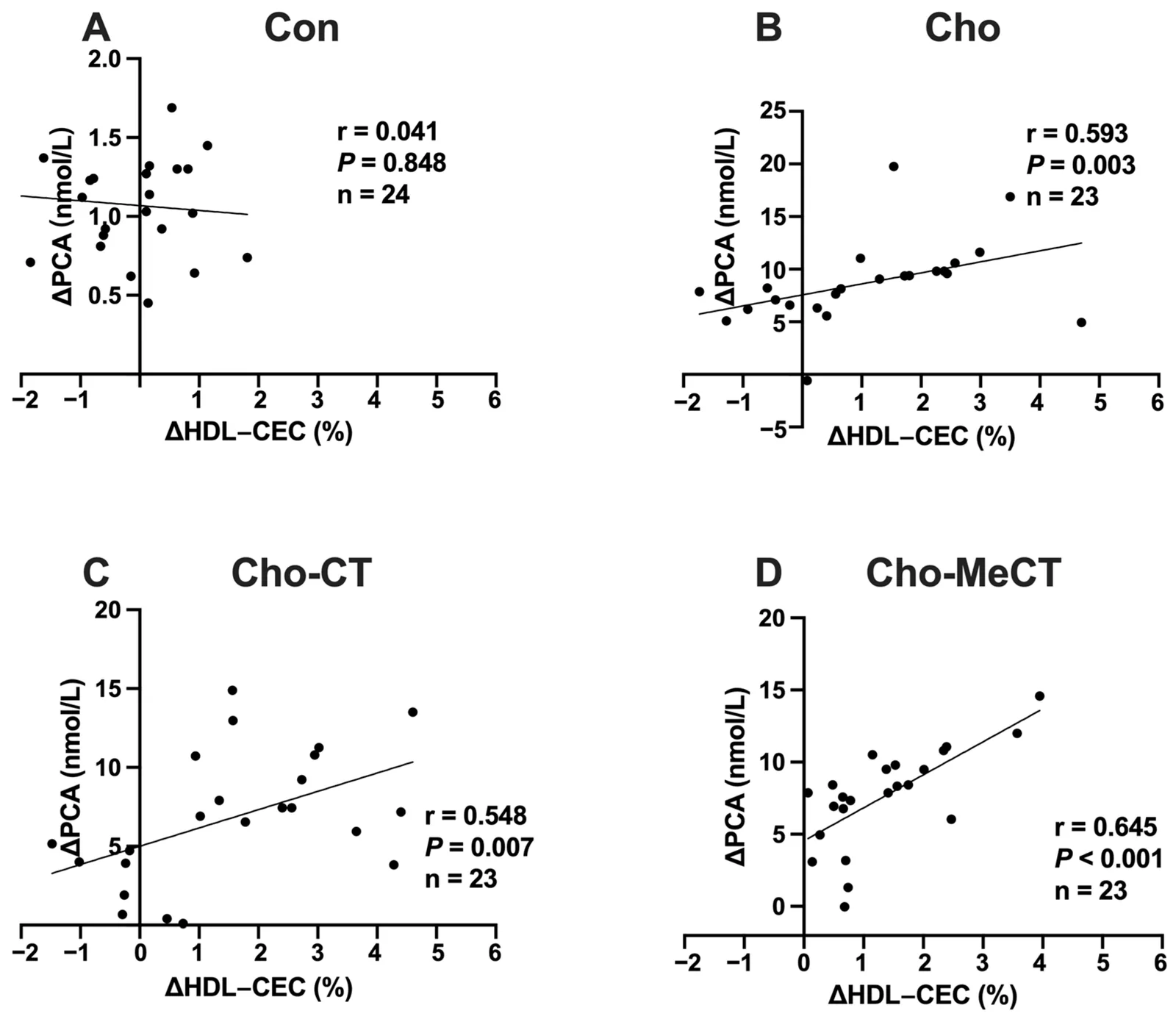
Correlation between changes in HDL-CEC from macrophages and plasma protocatechuic acid. The relation between the changes in HDL-CEC from macrophages and the changes in the concentrations of plasma of protocatechuic acid in the Con (**A**), Cho (**B**), Cho-CT (**C**), Cho-MeCT (**D**) groups. The values deviated from normality and were analyzed by Spearman’s rank correlation coefficients (*r_s_*). Cho, Brussels chicory; Cho-CT, Brussels chicory supplementation combined with supervised concurrent resistance exercise and high-intensity interval training; Cho-MeCT, mindfulness-enhanced concurrent resistance exercise and high-intensity interval training with Brussels chicory; Con, control; HDL-CEC, high-density lipoprotein cholesterol efflux capacity; PCA, protocatechuic acid. *p* values were adjusted using Bonferroni correction (threshold: *p* < 0.0125).

As shown in Table 3, Tables S8 and S9, the group-by-time interaction for HDL-CEC from macrophages reached statistical significance (*p* < 0.001), indicating that the overall trajectories of plasma HDL-CEC from macrophage changes differed significantly across the four tested groups. Pairwise comparisons with Bonferroni correction further revealed that at week 8, the levels of HDL-CEC from macrophages in the Cho, Cho-CT, and Cho-MeCT groups were all significantly higher than those in the Con group [Cho vs. Con: difference, 1.18 (95% CI: 0.40 to 1.97), *p* < 0.001; Cho-CT vs. Con: difference, 1.78 (95% CI: 0.77 to 2.80), *p* < 0.001; Cho-MeCT vs. Con: difference, 1.52 (95% CI: 0.81 to 2.23), *p* < 0.001; Table 3], suggesting that all three interventions increased plasma HDL-CEC from macrophages at the end of the intervention period. No significant differences in terms of HDL-CEC from macrophages were observed among the three intervention groups (Table S9), suggesting that all three interventions increased plasma HDL-CEC from macrophages to the comparable extents at the end of the intervention period. Of note, the correlation between changes in HDL-CEC from macrophage and changes in protocatechuic acid was assessed using Bonferroni correction (threshold: *p* < 0.0125 for four comparisons across the four groups). As shown in Figure 2A, no significant association was observed in the Con group. However, positive correlations were found in the Cho, Cho-CT, and Cho-MeCT groups (Cho: r = 0.593, 95% CI [0.128, 0.898], *p* = 0.003; Cho-CT: r = 0.548, 95% CI [0.162, 0.777], *p* = 0.007; Cho-MeCT: r = 0.645, 95% CI [0.267, 0.880], *p* < 0.001; Figure 2B–D). Mediation analyses further showed that body weight change did not significantly mediate the improvement in HDL-CEC from macrophages induced by the interventions (Table S10).

### 3.3. Secondary Outcomes of HDL Subclass Profiles

Compared with the Con group, the Cho, Cho-CT, and Cho-MeCT groups all showed significantly reduced plasma pre-β-HDL levels at week 8 [Cho vs. Con: difference, −63.63 (95% CI: −84.94 to −42.31), *p* < 0.001; Cho-CT vs. Con: difference, −49.39 (95% CI: −69.31 to −29.48), *p* < 0.001; Cho-MeCT vs. Con: difference, −55.98 (95% CI: −76.31 to −35.66), *p* < 0.001; Table 3]. Similar to the changes in plasma pre-β-HDL, plasma HDL_3_ levels significantly decreased in all three intervention groups compared with the Con group [Cho vs. Con: difference, −133.84 (95% CI: −214.06 to −53.62), *p* = 0.001; Cho-CT vs. Con: difference, −120.68 (95% CI: −196.85 to −44.52), *p* = 0.002; Cho-MeCT vs. Con: difference, −139.07 (95% CI: −217.59 to −60.54), *p* < 0.001; Table 3]. Plasma HDL_2_ levels at week 8 were significantly higher in the Cho, Cho-CT, and Cho-MeCT groups compared with the control group [Cho vs. Con: difference, 251.00 (95% CI: 102.07 to 399.03), *p* < 0.001; Cho-CT vs. Con: difference, 297.01 (95% CI: 98.16 to 495.86), *p* < 0.001; Cho-MeCT vs. Con: difference, 246.84 (95% CI: 130.81 to 362.87), *p* < 0.001; Table 3]. No significant differences were observed among the three intervention groups for pre-β-HDL, HDL_2_, or HDL_3_ at either week 4 or week 8 (Table S9). In addition, no significant differences were observed between any intervention group and the control group for plasma lipids and apolipoproteins (TC, TG, HDL-C, LDL-C, and Apo A-I) (Table 4).

### 3.4. Correlation Between Changes in HDL-CEC from Macrophages and HDL Subclasses

Exploratory correlation analyses were performed to examine whether intervention-associated changes in HDL-CEC from macrophages were correlated with concurrent changes in HDL-related parameters. All correlation analyses were adjusted for multiple testing using Bonferroni correction. No significant correlations were observed between changes in HDL-CEC from macrophages and changes in pre-β-HDL, HDL_3_, or HDL_2_ in the Con group. In contrast, in the Cho, Cho-CT, and Cho-MeCT groups, changes in HDL-CEC from macrophages were positively correlated with changes in HDL_2_ (Cho: r = 0.611, 95% CI [0.260, 0.829], *p* = 0.002; Cho-CT: r = 0.590, 95% CI [0.141, 0.861], *p* = 0.003; Cho-MeCT: r = 0.579, 95% CI [0.155, 0.868], *p* = 0.004). However, the correlations between changes in HDL-CEC from macrophages and changes in pre-β-HDL (Cho: r = −0.382, 95% CI [−0.719, 0.074], *p* = 0.072; Cho-CT: r = −0.530, 95% CI [−0.827, −0.062], *p* = 0.009; Cho-MeCT: r = −0.525, 95% CI [−0.798, −0.126], *p* = 0.010) or HDL_3_ (Cho: r = −0.112, 95% CI [−0.565, 0.397], *p* = 0.612; Cho-CT: r = −0.328, 95% CI [−0.716, 0.177], *p* = 0.126; Cho-MeCT: r = −0.360, 95% CI [−0.749, 0.159], *p* = 0.092) did not reach statistical significance.

## 4. Discussion

Overweight and obesity, while largely preventable, remain among the most pressing public health challenges [1]. Improving overweight- and obesity-elicited complications, including dysregulated HDL function and subclass profiles even in the absence of clinically significant weight loss, has great clinical relevance. Dietary modulation and regular physical exercise are well-recognized lifestyle approaches for modulating HDL quantity and quality, yet the optimization of integrated diet–exercise protocols remains an unmet research need [41,42,43]. Our research group previously demonstrated that one week of Brussels chicory supplementation effectively enhances HDL-CEC from aortic endothelial cells in overweight adults (38.5 ± 6.2 years old) [23]. Extending the intervention duration from 1 week to 8 weeks via a pilot randomized controlled trial in relatively younger overweight adults (18.3 ± 1.7 years old), the present study further showed that chronic Brussels chicory supplementation does not affect adiposity but improves HDL-CEC from macrophages by 1.18% to 1.78% and induces favorable HDL subclass remodeling, with significant reductions in pre-β-HDL and HDL_3_ and a marked increase in HDL_2_. Interestingly, combined intervention with supervised conventional or mindfulness-enhanced concurrent exercise training (resistance exercise plus HIIT) failed to modulate the effects of Brussels chicory supplementation on HDL function and subclass profiles. This intriguing phenomenon strongly implies that Brussels chicory intervention and concurrent exercise training may modulate HDL function via overlapping underlying molecular mechanisms.

Because our study constitutes a four-arm pilot randomized controlled trial rather than a full 3 × 3 × 3 factorial trial, we cannot reliably attribute the observed improvements in HDL-CEC from macrophages and HDL subclass profiles to any individual intervention component, nor can we confirm bona fide synergistic effects. Nevertheless, our findings allow us to provide proof-of-concept that Brussels chicory-containing interventions can modulate HDL-related phenotypes in this pilot trial. We therefore call for future factorial trials with single-intervention arms, which would allow rigorous disentanglement of whether favorable outcomes arise predominantly from Brussels chicory supplementation, exercise, or mindfulness acting alone, or from genuine additive or synergistic interactions among these three components.

HDL-CEC from macrophages exhibits an inverse correlation with both prevalent and incident ASCVD, and this association remains independent of traditional risk factors including HDL cholesterol levels [4]. A randomized, crossover, controlled trial enrolling healthy European male volunteers demonstrated that 3-week dietary supplementation with high-polyphenol raw olive oil (366 mg/kg polyphenols) elevated HDL-CEC from macrophages by 3.05%, compared with low-polyphenol olive oil (2.7 mg/kg polyphenols) [44]. Another clinical investigation further validated that 3-week intake of functional virgin olive oil fortified with phenolic compounds (total polyphenol content: 500 mg/kg) significantly improved HDL-CEC from macrophages in hypercholesterolemic adults, relative to conventional virgin olive oil (80 mg/kg polyphenols) [45]. Additionally, dietary intervention with purified anthocyanin polyphenols (80–320 mg/kg) has been documented to favorably elevate HDL cholesterol levels and enhance HDL-CEC from macrophages in subjects with dyslipidemia [21]. It should be borne in mind that non-significant alterations in HDL-CEC from macrophages following supplementation with polyphenol-rich olive oil or anthocyanins were also reported [46,47]. Despite these inconsistent findings, existing evidence supports the concept that dietary intervention is a critical modulator for improving HDL functional properties, which confers protective benefits for cardiovascular health [11].

Of note, our current study showed that 8-week dietary supplementation with Brussels chicory (~110 mg phenolic acids) increased HDL-CEC from macrophages by 1.18%. Epidemiological data indicate that each 0.1% increment in HDL-CEC from macrophages correlates with a 5% reduction in the risk of adverse cardiovascular events [48,49]. Accordingly, the 1.18% elevation in HDL-CEC from macrophages induced by Brussels chicory supplementation in our study is estimated to reduce the risk of adverse cardiovascular events by approximately 59%, representing a clinically meaningful and promising cardioprotective effect. Future randomized controlled trials are warranted to clarify the long-term and dose–response impacts of Brussels chicory intervention on HDL-CEC from macrophages and the risk of adverse cardiovascular events in overweight populations.

HDL is a heterogeneous class of particles that could be simply classified into discoidal pre-β-HDL, medium spherical HDL_3_ and large spherical alpha HDL_2_ [50]. Differences in HDL subclasses have been documented to have varied abilities to remove cellular cholesterol, which may explain variable correlations between HDL-cholesterol and ASCVD risk [51]. For example, HDL_2_ particles display stronger abilities than their pre-β-HDL and HDL_3_ to increase HDL-CEC from macrophages [52]. Indeed, subjects with overweight or obesity often have a higher pre-β-HDL and HDL_3_ and also a lower HDL_2_, which might hamper HDL functions and, in turn, increase the risk of ASCVD [5]. Regarding our study, we observed that Brussels chicory supplementation for 8 weeks appreciably inhibited the overweight-elicited increments in pre-β-HDL and HDL_3_ and the reduction in HDL_2_, though this administration did not affect HDL cholesterol and apolipoprotein A-I concentrations. More importantly, our correlation analyses further showed that after Brussels chicory supplementation, the changes in HDL-CEC from macrophages correlated positively with the changes in HDL_2_, whereas weak negative correlations were observed for pre-β-HDL and HDL_3_, though these associations did not reach statistical significance. Collectively, these findings thus allow us to propose that the beneficial effect of chronic Brussels chicory supplementation on HDL-CEC from macrophages might be in part secondary to remodeling HDL subclasses.

The changes of HDL subclass after Brussels chicory supplementation prompted us to investigate the underlying mechanisms. HDL subclass profiles are highly dynamic and finely regulated by various factors, two of which are plasma LCAT and CETP [50]. LCAT esterifies free cholesterol located on the surface of pre-β-HDL to form cholesteryl esters which then partition into the lipoprotein core, resulting in the formation of HDL_3_ [53]; CETP exchanges cholesteryl esters from HDL_3_ to apolipoprotein B-containing lipoproteins (e.g., VLDL, LDL) for triglycerides [54], which generates large cholesteryl ester-poor and triglyceride-rich HDL_2_. Because Brussels chicory supplementation could not affect the abundance and enzymatic activities of LCAT and CETP, we favored the notion that neither LCAT nor CETP is involved in the effect of Brussels chicory on HDL subclass profiles. Future work is required to test whether other factors, such as hepatic lipase, endothelial lipase, lipoprotein lipase, hepatic and intestinal ATP-binding cassette transporter A1 and apolipoprotein A-I, factors known to affect HDL subclass profiles [50,55], are responsible for the Brussels chicory effect.

Regular exercise is able to increase HDL-CEC from macrophages and alter HDL subclass profiles with less pre-β-HDL and HDL_3_ and more HDL_2_ across diverse populations, including those with high risk of ASCVD and healthy athletes [41,56,57,58]. It thus seemed paradoxical that conventional or mindfulness-enhanced concurrent exercise training (resistance exercise plus HIIT) did not affect the effects of Brussels chicory on HDL-CEC functions and subclass profiles observed in the current study. Our current findings are reminiscent of a previous study that HDL-CEC from macrophages and HDL subclass profiles are not affected by a 24-week supervised resistance training in patients with peripheral artery disease [59]. Regarding the potential reasons for these unexpected findings, we propose several possibilities. One potential possibility is that the participants in the current study are relatively young (18.3 ± 1.7 years old) and have a functional HDL and also ideal HDL subclass profiles, though they are sedentary and overweight. Indeed, any tested parameters, such as plasma lipid and lipoprotein levels, are in acceptable ranges for physiological variables. On the other hand, the effects of Brussels chicory supplementation on HDL-CEC from macrophages and HDL subclass profiles are reaching the maximum, leaving little room for further improvement. Additionally, the duration for concurrent exercise training may be too short to elicit the changes in HDL metrics. Indeed, the amount of exercise but not exercise intensity might play a critical role in regulation of HDL metrics in humans [60].

This study has several limitations to consider. First, although significant correlations exist between HDL-CEC from macrophages and HDL subclass profiles across the Cho, Cho-CT, and Cho-MeCT groups, causal relationships remain speculative and cannot be confirmed based on the present findings. Second, young overweight male college students recruited from a single university tended to be highly self-motivated. This is reflected by the fact that only one participant withdrew from each study arm throughout the trial, and participants maintained consistent habitual dietary and physical-activity patterns outside of supervised exercise sessions. While our observations provide preliminary evidence regarding the effects of multi-component interventions (Brussels chicory alone, Brussels chicory combined with supervised concurrent resistance exercise plus high-intensity interval training or mindfulness-augmented concurrent resistance exercise and high-intensity interval training) on HDL function within this specific subgroup, we caution against direct generalization to broader populations. Future multi-site trials recruiting more demographically diverse participants (e.g., women, middle-aged and older adults, as well as community-dwelling overweight or obese populations) are needed to validate whether these intervention effects can be replicated across different demographic subgroups. Third, Brussels chicory alone, or Brussels chicory combined with supervised concurrent exercise training, altered HDL-CEC from macrophages and HDL subclass profiles; however, it remains unknown whether these interventions affect other proposed atheroprotective properties of HDL, such as anti-oxidation, anti-inflammation or inhibition of thrombosis [50,51]. Fourth, although our association analysis indicated that protocatechuic acid partially accounts for the elevated HDL-CEC from macrophages induced by Brussels chicory supplementation, causal relationships cannot be confirmed at present. As a whole food, Brussels chicory contains abundant nutrients and numerous bioactive compounds, including phenolic acids (chicoric acid, chlorogenic acid, protocatechuic acid, gallic acid), flavonoids, and terpenoids [23,38]. Therefore, apart from protocatechuic acid, other uncharacterized phytochemicals in Brussels chicory may synergistically mediate its beneficial effects on HDL-CEC from macrophages. Fifth, the change-score correlation analyses were not adjusted for baseline values, which may have introduced potential bias in estimating the relationships between changes in variables. Finally, it remains unknown whether an alteration in HDL-CEC from macrophages and HDL subclass profiles elicited by Brussels chicory alone, or Brussels chicory combined with supervised concurrent exercise training in overweight adults, reduces the risk of developing cardiovascular disease and warrants further evaluation.

## 5. Conclusions

To our knowledge, this randomized controlled trial provides direct evidence for the impact of Brussels chicory combined with supervised concurrent exercise training (resistance exercise plus HIIT) integrated with or without mindfulness on HDL function and subclass profiles in overweight college students. Our preliminary findings have shown that dietary Brussels chicory supplementation for 8 weeks is able to modestly increase HDL-CEC from macrophages and also remodel HDL subclass profiles with less pre-β-HDL and HDL_3_ and more HDL_2_, all of which could not be further affected by a co-treatment with concurrent training with unknown mechanisms. These observations thus suggest that dietary supplementation of Brussels chicory with or without concurrent exercise might be a promising approach for increasing HDL function in young adults with overweight.

## Figures and Tables

**Table 1:** Baseline characteristics of the study participants (*n* = 93).

Index	Con (*n* = 24)	Cho (*n* = 23)	Cho-CT (*n* = 23)	Cho-MeCT (*n* = 23)	*p*
Age, years	18.08 ± 0.28	18.78 ± 2.88	18.39 ± 1.70	18.09 ± 0.29	0.441
Height, cm	175.28 ± 5.49	173.42 ± 6.52	173.84 ± 6.98	175.83 ± 4.98	0.481
Weight, kg	83.48 ± 11.52	84.78 ± 12.43	80.20 ± 8.26	87.10 ± 12.81	0.229
BMI, kg/m^2^	27.08 ± 2.69	28.19 ± 3.96	26.51 ± 1.94	28.11 ± 3.44	0.197
Percentage of body fat, %	23.64 ± 2.90	24.64 ± 3.25	22.86 ± 2.50	24.90 ± 2.83	0.069
Waist circumference, cm	93.00 ± 7.11	95.54 ± 10.06	90.00 ± 7.78	95.59 ± 8.18	0.081
Hip circumference, cm	105.54 ± 5.04	106.48 ± 7.17	103.35 ± 4.45	107.44 ± 6.13	0.106
Vital capacity, mL	4932.00 ± 809.22	4724.78 ± 774.66	4652.83 ± 685.11	4776.35 ± 745.27	0.630
Health-related physical fitness	250.58 ± 33.08	245.52 ± 34.12	261.50 ± 35.32	242.37 ± 30.73	0.233
Exercise activity level, MET-min/w	1421.34 ± 286.47	1407.24 ± 249.62	1457.29 ± 210.48	1446.59 ± 206.37	0.890
Mindfulness	64.71 ± 9.72	65.48 ± 10.71	62.52 ± 8.61	60.83 ± 9.86	0.358
Energy, kcal/d	2125.47 ± 260.26	2082.70 ± 230.36	2196.31 ± 207.81	2155.23 ± 283.90	0.462
Carbohydrate, kcal/d	1195.10 ± 180.48	1173.03 ± 170.47	1280.57 ± 133.09	1276.93 ± 233.75	0.103
Protein, kcal/d	326.27 ± 30.88	323.53 ± 33.01	325.69 ± 31.69	302.71 ± 36.58	0.051
Fat, kcal/d	604.10 ± 59.30	586.14 ± 50.04	590.05 ± 55.58	575.60 ± 50.50	0.345
Dietary fiber, kcal/d	20.59 ± 3.64	20.02 ± 4.94	21.01 ± 5.33	18.54 ± 3.27	0.244
TC, mmol/L	4.35 ± 0.44	4.55 ± 0.46	4.47 ± 0.62	4.45 ± 0.46	0.602
TG, mmol/L	1.18 ± 0.19	1.15 ± 0.21	1.12 ± 0.17	1.15 ± 0.22	0.803
LDL-C, mmol/L	2.21 ± 0.26	2.29 ± 0.28	2.35 ± 0.24	2.27 ± 0.23	0.305
HDL-C, mmol/L	1.23 ± 0.27	1.29 ± 0.33	1.24 ± 0.28	1.17 ± 0.24	0.525
Apo A-I, mg/L	1119.40 ± 245.51	1161.67 ± 296.99	1173.71 ± 246.59	1071.71 ± 229.48	0.524

Data are mean ± SD. Apo A-I, Apolipoprotein A-I; BMI, body mass index; Cho, Brussels chicory; Cho-CT, Brussels chicory supplementation combined with supervised concurrent resistance exercise and high-intensity interval training; Cho-MeCT, mindfulness-enhanced concurrent resistance exercise and high-intensity interval training with Brussels chicory; Con, control; HDL-C, high-density lipoprotein cholesterol; LDL-C, low-density lipoprotein cholesterol; MET-min/w, metabolic equivalent of task-minutes per week; TC, total cholesterol; TG, triglyceride.

**Table 2:** Compliance of the study participants to different interventions *.

Index	Con (*n* = 24)	Cho (*n* = 23)	Cho-CT (*n* = 23)	Cho-MeCT (*n* = 23)	G × T Effect	Cho-MeCT vs. Con		Cho-CT vs. Con		Cho vs. Con	
						MD (95% CI)	*p*	MD (95% CI)	*p*	MD (95% CI)	*p*
Weight, kg											
Baseline	83.48 ± 11.52	84.78 ± 12.43	80.204 ± 8.26	87.10 ± 12.81	**<0.001**						
Week 8	83.10 ± 10.85	84.72 ± 12.71	78.83 ± 7.41	84.60 ± 12.44		−2.04 (−3.57 to −0.51)	**0.003**	−0.62 (−1.95 to 0.70)	1.00	0.34 (−0.97 to 1.66)	1.00
BMI, kg/m^2^											
Baseline	27.08 ± 2.69	28.19 ± 3.96	26.52 ± 1.94	28.11 ± 3.44	**0.020**						
Week 8	26.95 ± 2.52	28.20 ± 4.06	26.28 ± 2.11	27.42 ± 3.42		−0.54 (−1.07 to −0.02)	**0.036**	−0.10 (−0.55 to 0.35)	1.00	0.16 (−0.39 to 1.07)	1.00
Vital capacity, mL											
Baseline	4932.00 ± 809.22	4724.78 ± 774.66	4652.83 ± 685.11	4776.35 ± 745.27	**<0.001**						
Week 8	5012.17 ± 801.59	4862.3 ± 774.80	5502.3 ± 526.28	5466.43 ± 683.52		593.68 (325.76 to 861.59)	**<0.001**	740.18 (423.20 to 1057.16)	**<0.001**	35.73 (−180.84 to 252.30)	1.00
HRPF											
Baseline	250.56 ± 33.08	245.52 ± 34.12	261.50 ± 35.23	242.36 ± 30.74	**<0.001**						
Week 8	233.66 ± 33.45	230.30 ± 33.12	277.13 ± 29.53	259.62 ± 29.49		33.65 (24.19 to 43.11)	**<0.001**	33.23 (22.54 to 43.92)	**<0.001**	1.38 (−8.00 to 10.75)	1.00
Mindfulness											
Baseline	64.71 ± 9.72	65.48 ± 10.71	62.52 ± 8.61	60.83 ± 9.86	**<0.001**						
Week 8	65.88 ± 9.66	65.61 ± 10.95	64.00 ± 11.41	75.35 ± 11.44		12.26 (5.46 to 19.05)	**<0.001**	−0.31 (−6.94 to 6.33)	1.00	−0.82 (−6.99 to 5.35)	1.00
PCA, nmol/L											
Baseline	5.87 ± 1.72	5.44 ± 2.14	6.11 ± 2.44	5.94 ± 2.16	**<0.001**						
Week 8	6.94 ± 1.77	14.13 ± 4.72	13.11 ± 4.36	13.58 ± 3.52		6.58 (4.76 to 8.41)	**<0.001**	5.97 (3.76 to 8.17)	**<0.001**	7.54 (5.35 to 9.74)	**<0.001**

* Data are mean ± SD. Analyses were performed using generalized estimating equations (GEE). For all outcomes presented in this table, covariates included baseline measurement values, time, group, and group-by-time interaction. Pairwise comparisons were performed using linear contrasts with Bonferroni correction. Boldfaced *p* values denote significance after correction. BMI, body mass index; Cho, Brussels chicory; Cho-CT, Brussels chicory supplementation combined with supervised concurrent resistance exercise and high-intensity interval training; Cho-MeCT, mindfulness-enhanced concurrent resistance exercise and high-intensity interval training with Brussels chicory; Con, control; HRPF, health-related physical fitness; PCA, protocatechuic acid; G × T, group-by-time interaction effect; MD, mean difference; CI, confidence intervals.

**Table 3:** Effects of different interventions on primary and secondary outcomes *.

Index	Con (*n* = 24)	Cho (*n* = 23)	Cho-CT (*n* = 23)	Cho-MeCT (*n* = 23)	G × T Effect	Cho-MeCT vs.Con		Cho-CT vs. Con		Cho vs. Con	
						MD (95% CI)	*p*	MD (95% CI)	*p*	MD (95% CI)	*p*
HDL-CEC, %											
Baseline	8.13 ± 1.73	7.85 ± 2.20	8.08 ± 1.76	8.06 ± 1.39	**<0.001**						
Week 8	7.93 ± 1.49	8.93 ± 1.36	9.67 ± 2.01	9.42 ± 1.28		1.52 (0.81 to 2.23)	**<0.001**	1.78 (0.77 to 2.80)	**<0.001**	1.18 (0.40 to 1.97)	**<0.001**
Pre-*β*-HDL, mg/L											
Baseline	112.44 ± 32.20	120.69 ± 33.68	115.19 ± 28.28	107.95 ± 22.69	**<0.001**						
Week 8	122.15 ± 27.03	62.46 ± 15.80	74.07 ± 15.80	64.03 ± 22.83		−55.98 (−76.31 to −35.66)	**<0.001**	−49.40 (−69.31 to −29.48)	**<0.001**	−63.63 (−84.94 to −42.32)	**<0.001**
Pre-*β*_1_-HDL, mg/L											
Baseline	66.09 ± 18.85	69.12 ± 22.20	67.51 ± 17.56	64.09 ± 15.29	**<0.001**						
Week 8	68.61 ± 21.54	34.37 ± 14.10	40.57 ± 10.67	34.07 ± 13.85		−33.57 (−47.24 to −19.89)	**<0.001**	−28.74 (−42.44 to −15.03)	**<0.001**	−35.72 (−50.55 to −20.88)	**<0.001**
Pre-*β*_2_-HDL, mg/L											
Baseline	46.35 ± 14.34	51.57 ± 19.13	47.68 ± 14.79	43.86 ± 11.28	**<0.001**						
Week 8	53.54 ± 10.66	28.09 ± 12.81	33.51 ± 9.22	29.96 ± 13.16		−22.45 (−32.30 to −12.60)	**<0.001**	−20.65 (−29.67 to −11.62)	**<0.001**	−27.84 (−38.42 to −17.26)	**<0.001**
HDL_3_, mg/L											
Baseline	550.72 ± 113.58	576.86 ± 134.88	573.03 ± 140.83	538.40 ± 115.86	**<0.001**						
Week 8	561.51 ± 119.43	442.17 ± 88.30	453.20 ± 93.03	415.61 ± 121.63		−139.07 (−217.59 to −60.54)	**<0.001**	−120.68 (−196.85 to −44.52)	**<0.001**	−133.84 (−214.06 to −53.62)	**<0.001**
HDL_3c_, mg/L											
Baseline	79.04 ± 20.30	81.79 ± 22.16	85.29 ± 24.62	84.30 ± 19.06	0.958						
Week 8	82.60 ± 20.10	86.05 ± 18.40	90.18 ± 24.96	93.58 ± 19.81		7.84 (−5.92 to 21.60)	0.796	3.85 (−13.32 to 21.02)	1.00	1.81 (−13.71 to 17.32)	1.00
HDL_3b_, mg/L											
Baseline	241.02 ± 60.51	249.44 ± 65.63	249.52 ± 75.24	232.57 ± 56.82	**<0.001**						
Week 8	249.32 ± 74.33	157.46 ± 52.60	172.74 ± 81.91	148.84 ± 72.68		−95.60 (−146.85 to −44.36)	**<0.001**	−81.48 (−133.70 to −29.26)	**<0.001**	−96.71 (−147.88 to −45.54)	**<0.001**
HDL_3a_, mg/L											
Baseline	230.67 ± 65.40	245.63 ± 92.34	238.21 ± 70.55	221.53 ± 62.95	**0.030**						
Week 8	229.60 ± 58.01	198.66 ± 34.20	190.28 ± 48.43	173.19 ± 39.14		−51.94 (−84.34 to −19.53)	**<0.001**	−43.00 (−79.83 to −6.17)	**0.012**	−38.26 (−79.40 to 2.88)	0.085
HDL_2_, mg/L											
Baseline	456.24 ± 125.92	464.12 ± 292.97	485.50 ± 216.74	425.36 ± 189.88	**<0.001**						
Week 8	471.23 ± 181.58	725.66 ± 139.03	781.01 ± 311.77	704.58 ± 129.20		246.84 (130.81 to 362.87)	**<0.001**	297.01 (98.16 to 495.86)	**<0.001**	251.00 (102.07 to 399.03)	**<0.001**
HDL_2a_, mg/L											
Baseline	259.86 ± 76.80	264.38 ± 81.64	265.84 ± 76.13	242.46 ± 80.89	**0.040**						
Week 8	257.11 ± 80.03	319.21 ± 76.09	329.37 ± 95.61	286.03 ± 82.49		38.36 (−8.82 to 85.55)	0.192	69.01 (13.00 to 125.02)	**0.007**	59.64 (0.40 to 118.88)	**0.047**
HDL_2b_, mg/L											
Baseline	196.38 ± 77.23	199.74 ± 242.01	219.65 ± 179.57	182.91 ± 150.71	**<0.001**						
Week 8	214.11 ± 122.25	406.46 ± 93.68	451.64 ± 237.04	418.55 ± 96.12		209.38 (119.30 to 299.46)	**<0.001**	229.00 (74.25 to 383.76)	**0.001**	191.12 (86.86 to 295.37)	**<0.001**
LCAT, nmol·L^−1^·h^−1^											
Baseline	113.81 ± 17.44	126.27 ± 14.25	123.12 ± 17.84	121.90 ± 16.26	0.350						
Week 8	128.35 ± 13.82	141.75 ± 17.82	129.02 ± 12.38	136.57 ± 16.82		5.21 (−7.81 to 18.23)	1.00	−2.79 (−14.23 to 8.64)	1.00	8.76 (−4.54 to 22.05)	0.493
CETP, nmol·L^−1^·h^−1^											
Baseline	87.84 ± 7.91	86.36 ± 8.12	85.34 ± 7.80	83.64 ± 9.31	0.536						
Week 8	90.90 ± 11.06	91.86 ± 9.78	90.55 ± 11.46	86.01 ± 9.58		−3.12 (−10.81 to 4.56)	1.00	0.70 (−8.13 to 9.53)	1.00	1.59 (−6.14 to 9.31)	1.00

* Data are mean ± SD. Analyses were performed using generalized estimating equations (GEE). For the primary outcome (HDL-CEC), covariates included baseline measurement values, time, group, group-by-time interaction, and baseline body mass index (BMI). For the other outcomes, covariates included baseline measurement values, time, group, and group-by-time interaction (without baseline BMI adjustment). Pairwise comparisons were performed using linear contrasts with Bonferroni correction. Boldfaced *p* values denote significance after correction. CETP, cholesteryl ester transfer protein; Cho, Brussels chicory; Cho-CT, Brussels chicory supplementation combined with supervised concurrent resistance exercise and high-intensity interval training; Cho-MeCT, mindfulness-enhanced concurrent resistance exercise and high-intensity interval training with Brussels chicory; Con, control; HDL-CEC, high-density lipoprotein cholesterol efflux capacity; LCAT, lecithin: cholesterol acyltransferase; G × T, group-by-time interaction effect; MD, mean difference; CI, confidence interval.

**Table 4:** Effects of different intervention on blood lipid parameters *.

Index	Con (*n* = 24)	Cho (*n* = 23)	Cho-CT (*n* = 23)	Cho-MeCT (*n* = 23)	G × T Effect	Cho-MeCT vs. Con		Cho-CT vs. Con		Cho vs. Con	
						MD (95% CI)	*p*	MD (95% CI)	*p*	MD (95% CI)	*p*
TC, mmol/L											
Baseline	4.35 ± 0.44	4.55 ± 0.46	4.47 ± 0.62	4.45 ± 0.46	0.056						
Week 8	4.34 ± 0.53	4.47 ± 0.37	4.23 ± 0.41	4.18 ± 0.41		−0.23 (−0.47 to 0.003)	**0.045**	−0.20 (−0.42 to 0.01)	0.073	−0.02 (−0.30 to 0.26)	1.00
TG, mmol/L											
Baseline	1.18 ± 0.19	1.15 ± 0.21	1.12 ± 0.17	1.15 ± 0.22	0.413						
Week 8	1.20 ± 0.15	1.19 ± 0.15	1.11 ± 0.12	1.14 ± 0.14		−0.05 (−0.16 to 0.05)	1.00	−0.07 (−0.16 to 0.02)	0.187	−0.00 (−0.11 to 0.11)	1.00
LDL-C, mmol/L											
Baseline	2.21 ± 0.26	2.29 ± 0.28	2.35 ± 0.24	2.27 ± 0.23	0.108						
Week 8	2.15 ± 0.45	2.19 ± 0.29	2.22 ± 0.31	2.16 ± 0.16		−0.02 (−0.30 to 0.26)	1.00	−0.001 (−0.33 to 0.32)	1.00	0.002 (−0.30 to 0.31)	1.00
HDL-C, mmol/L											
Baseline	1.23 ± 0.27	1.29 ± 0.33	1.24 ± 0.28	1.17 ± 0.24	0.245						
Week 8	1.23 ± 0.25	1.28 ± 0.17	1.30 ± 0.25	1.21 ± 0.16		0.02 (−0.10 to 0.14)	1.00	0.07 (−0.07 to 0.222)	1.00	0.02 (−0.14 to 0.18)	1.00
Apo A-I, mg/L											
Baseline	1119.40 ± 245.51	1161.97 ± 297.00	1173.71 ± 246.59	1071.71 ± 229.48	0.352						
Week 8	1154.89 ± 295.06	1230.30 ± 185.63	1308.29 ± 300.86	1184.22 ± 229.03		60.36 (−86.74 to 207.45)	1.00	118.06 (−69.36 to 305.47)	0.579	47.91 (−139.88 to 235.69)	1.00

* Data are mean ± SD. Analyses were performed using generalized estimating equations (GEE). Covariates included baseline measurement values, time, group, and group-by-time interaction. Pairwise comparisons were performed using linear contrasts with Bonferroni correction. Boldfaced *p* values denote significance after correction. Apo A-I, Apolipoprotein A-I; Cho, Brussels chicory; Cho-CT, Brussels chicory supplementation combined with supervised concurrent resistance exercise and high-intensity interval training; Cho-MeCT, mindfulness-enhanced concurrent resistance exercise and high-intensity interval training with Brussels chicory; Con, control; HDL-C, high-density lipoprotein cholesterol; LDL-C, low-density lipoprotein cholesterol; TC, total cholesterol; TG, triglyceride; G × T, group-by-time interaction effect; MD, mean difference; CI, confidence interval.

## Data Availability

The original contributions presented in this study are included in the article/Supplementary Materials. Further inquiries can be directed to the corresponding author.

## Supplementary Materials

The following supporting information can be downloaded at https://www.mdpi.com/article/10.3390/nu18162678/s1, Table S1: Composition and nutritional profile of Brussels chicory; Table S2: Supervised concurrent exercise training protocol; Table S3: Supervised exercise pool; Table S4: Mindfulness intervention protocol; Table S5: Comparison of single-intervention processes and times between Cho-MeCT and Cho-CT groups; Table S6: Effects of different interventions on anthropometric measurements, vital capacity, HRPF, mindfulness, dietary nutrient intakes, and serum parameters; Table S7: Comparisons of Cho-MeCT, Cho-CT, and Cho on anthropometric measurements, vital capacity, HRPF, mindfulness, dietary nutrient intakes, and serum parameters; Table S8: Effects of different interventions on primary and secondary outcomes; Table S9: Comparisons of Cho-MeCT, Cho-CT, and Cho on primary and secondary outcomes; Table S10: Mediation analysis of body weight change in the pathway from group assignment to HDL-CEC change.

## Abbreviations

The following abbreviations are used in this manuscript:

Apo A-I	Apolipoprotein A-I
ASCVD	Atherosclerotic cardiovascular disease
BMI	Body mass index
CETP	Cholesteryl ester transfer protein
Cho	Brussels chicory
Cho-CT	Brussels chicory supplementation combined with supervised concurrent resistance exercise and high-intensity interval training
Cho-MeCT	Mindfulness-enhanced concurrent resistance exercise and high-intensity interval training with Brussels chicory
HDL-C	High-density lipoprotein cholesterol
HDL-CEC	High-density lipoprotein cholesterol efflux capacity
LDL-C	Low-density lipoprotein cholesterol
LCAT	Lecithin: cholesterol acyltransferase
TC	Total cholesterol
TG	Triglyceride
