# Supplementary material for: Effects of Brussels Chicory and Concurrent Exercise Training on HDL Function and Subclass Profiles in Overweight College Students: A Pilot Randomized Controlled Trial
Source: Nutrients. 2026 Aug 16;18(16):2678. doi: 10.3390/nu18162678 (PMC13516577; doi:10.3390/nu18162678)
Supplement: Supplementary file 1 [file nutrients-18-02678-s001.zip › Table S1.pdf]

Table S1. Composition and nutritional profile of Brussels chicory*

Item	Brussels chicory (Per 100 g)
Energy, kcal	19.60
Protein, g	1.04
Fat, g	0.25
Carbohydrate, g	3.50
Crude fiber, g	1.22
Moisture, g	91.21
Total phenolic acids, mg	221.32
Protocatechuic acid, mg	33.72
Gallic acid, mg	24.38
Caffeic acid, mg	9.32
5-Caffeoylquinic acid, mg	98.25
Caftaric acid, mg	20.17
Chicoric acid, mg	35.48

*Nutritional ingredient values for Brussels chicory were referenced to the USDA National Nutrient Database. The identification and quantification of phenolic acids in Brussels chicory were performed via high-performance liquid chromatography, following our previously published protocol (Food Sci Nutr. 2019.7(9):3071-3080).
