# Supplementary material for: Effects of Brussels Chicory and Concurrent Exercise Training on HDL Function and Subclass Profiles in Overweight College Students: A Pilot Randomized Controlled Trial
Source: Nutrients. 2026 Aug 16;18(16):2678. doi: 10.3390/nu18162678 (PMC13516577; doi:10.3390/nu18162678)
Supplement: Supplementary file 1 [file nutrients-18-02678-s001.zip › Table S10.pdf]

Table S10. Mediation analysis of body weight change in the pathway from group assignment to HDL-CEC change

Comparison	Total effect (c)	Direct effect (c')	Indirect effect (a×b)	BootSE	95% Bootstrap CI	Mediation
Cho vs. Con	1.284**	1.315**	-0.030	0.067	(-0.173, 0.108)	Not significant
Cho-CT vs. Con	1.787***	1.734***	0.054	0.083	(-0.056, 0.271)	Not significant
Cho-MeCT vs. Con	1.555***	1.353**	0.202	0.179	(-0.101, 0.610)	Not significant

*Notes: Bootstrap resampling was performed with 5,000 iterations. Mediation was considered significant if the 95% CI did not include zero. *$P < 0.05$, **$P < 0.01$, **$P < 0.001$.

Cho, Brussels chicory; Cho-CT, Brussels chicory supplementation combined with supervised concurrent resistance exercise and high-intensity interval training; Cho-MeCT, mindfulness-enhanced concurrent resistance exercise and high-intensity interval training with Brussels chicory; Con, control; HDL-CEC, high-density lipoprotein cholesterol efflux capacity; BootSE, bootstrap standard error; CI, confidence interval.
