# Supplementary material for: Effects of Brussels Chicory and Concurrent Exercise Training on HDL Function and Subclass Profiles in Overweight College Students: A Pilot Randomized Controlled Trial
Source: Nutrients. 2026 Aug 16;18(16):2678. doi: 10.3390/nu18162678 (PMC13516577; doi:10.3390/nu18162678)
Supplement: Supplementary file 1 [file nutrients-18-02678-s001.zip › Table S2.pdf]

**Table S2.** Supervised concurrent exercise training protocol

| Training Phase | Intensity | Weeks 1-2 | Weeks 3-8 |
|---|---|---|---|
| Warm-up | 60%-70% HRmax | Jogging + Stretching | Jogging + Stretching |
| HIIT | 75%-85% HRmax | Low-volume HIIT: 30s × 8-20 movements; 0-15s rest between movements depending on intensity. | HIIT: 30s × 15-30 movements; 0-15s rest between movements depending on intensity. |
| RT | See right columns | 30%-50% 1RM, 6-9 movements, 12-20 repetitions per movement, 30-60s rest. | 30%-80% 1RM, 6-10 movements, 8-20 repetitions per movement, 30-60s rest. |
| Cool-down | - | Stretching | Stretching |
| Total Exercise Duration | | 60 min | |

Note: HIIT, high-intensity interval training; HRmax, maximum heart rate; RM, repetition maximum; RT, resistance Training.
