# Supplementary material for: Effects of Brussels Chicory and Concurrent Exercise Training on HDL Function and Subclass Profiles in Overweight College Students: A Pilot Randomized Controlled Trial
Source: Nutrients. 2026 Aug 16;18(16):2678. doi: 10.3390/nu18162678 (PMC13516577; doi:10.3390/nu18162678)
Supplement: Supplementary file 1 [file nutrients-18-02678-s001.zip › Table S3.pdf]

Table S3. Supervised exercise pool

Target Area	**HIIT (**Variations in brackets**)**	**RT (**Variations in brackets**)**
Upper body dominant	Dumbbell row, shoulder press (single-arm shoulder press), bent-arm chest fly, boxing (diagonal upward), push-ups (kneeling / close-grip / + inchworm), thrusters, bench press (close-grip), overhead press, triceps extensions (lying), bicep curls (kneeling)	Push-ups (kneeling / pseudo planche / kneeling diamond / explosive / close-grip), bicep curls, triceps extensions, bench dips, inverted rows (lying), shoulder press (arnold press), flies (bent-over), front raise, front press, military press, plate halos, bent-over row
Core dominant	Prone plank (low / high / high-reach / dynamic / rotation / shoulder tap), reverse plank, side plank, plank tucks (cross-body / jumping), mountain climbers (cross-body mountain climbers), crunches (reverse straight-leg), v-ups (single-leg / back extension), prone swimming, russian twist, lying ankle taps, bear crawl, w-extensions (hold), 45-degree leg hold, dumbbell swings, seated rapid knee taps, elbow-to-knee (+ toe touch), inchworm, side double-leg tucks	Crunches (reverse / reverse straight-leg), russian twist, plank knee-to-elbow (prone), bicycle crunches (reverse), back extensions, v-ups (cross-body / back extension), plank, side plank, dead bug, toe touches (lying), crawling, hanging leg raises, all back extension variations, scissor kicks
Lower body dominant	Walking lunges, kicks, side lunges, reverse lunges, curtsy lunges (jumping), squats (+ overhead reach / hold / rapid / jump / pulse / overhead / sumo / front squat), half squats (jumping), glute kickbacks, high knees (marching / with reach), jump rope, lateral agility ladder, lateral shuffle to vertical jump, small hops (lateral), hip rotation hops (squat jump), running in place, lateral knee tucks, tuck jumps, crab walk, standing hip abduction, glute bridge (single-leg / hold / marching), donkey kicks (prone), skater jumps (high / low), scissor jumps, step-up jumps, calf raises, shuttlecock kicks, lunge jumps	Squat (sumo / staggered / explosive-eccentric / front squat), half squat, clamshells, lunges (reverse lunges), kettlebell swings, glute bridge (single-leg / feet together), calf raises, deadlift (sumo / staggered / single-leg), leg raises (prone), power clean, leg curls, leg press

Table S3. Continued

**Target Area**	**HIIT (**Variations in brackets**)**	**RT (**Variations in brackets**)**
Composite move	Windmills, running in place, jumping jacks (t-jacks / plank jacks), hip rotations (hand supported), under-leg claps, burpees (half-rep), o-jacks (knee tuck jumps), star jumps, toe touch to overhead reach and rotation, sit-throughs, toe switches	Free combination of the above upper and lower body dominant movements

Note: The table lists individual movements only; in practice, two movements could be combined (e.g., Squat + Single-arm Shoulder Press) or performed with added weight. HIIT, high-intensity interval training; RT, resistance training.
