# Supplementary material for: Effects of Brussels Chicory and Concurrent Exercise Training on HDL Function and Subclass Profiles in Overweight College Students: A Pilot Randomized Controlled Trial
Source: Nutrients. 2026 Aug 16;18(16):2678. doi: 10.3390/nu18162678 (PMC13516577; doi:10.3390/nu18162678)
Supplement: Supplementary file 1 [file nutrients-18-02678-s001.zip › Table S4.pdf]

Table S4. Mindfulness intervention protocol

Week	Session 1	Session 2	Session 3
1	Mind–body connection; mindfulness of breath; mindful acceptance of exercise-induced fatigue, soreness, and discomfort	Breath awareness; mind–body connection and experience sharing	Mindful eating; mindfulness of breath and experience sharing
2	Body scan; mindfulness of breath and experience sharing	Acceptance (one of the nine attitudes of mindfulness); body scan and experience sharing	Beginner's mind (one of the nine attitudes); sharing experiences of applying the attitude of acceptance
3	Non-judging (one of the nine attitudes); sharing experiences of applying the beginner's mind attitude; body scan and experience sharing	Recognizing small joys in daily life; review and sharing of body scan experiences; the attitude of "non-judging"	Why smiles become fewer; discovering moments of joy; patience (one of the nine attitudes)
4	Unpleasant moments and loving-kindness meditation; letting go / non-attachment (one of the nine attitudes)	Sharing unpleasant moments and practicing acceptance; three-minute breathing space	Non-striving (one of the nine attitudes); breathing space / loving-kindness meditation practice and experience sharing
5	Mid-term review; sharing physical and mental changes	Trust (one of the nine attitudes); three body parts you are satisfied with	Gratitude and generosity (1) (one of the nine attitudes); mindfulness during strength training
6	Gratitude and generosity (2); noticing moments of gratitude and generosity	Mindfulness day; mindfulness during exercise	Mindfulness day sharing; bringing mindfulness into daily life
7	"Big game" meditation; mindfulness of breath and experience sharing	Mindfulness day; loving-kindness body scan and experience sharing	Mindfulness day sharing; loving-kindness body scan and experience sharing
8	"Big game" meditation; meditation practice and sharing	Partner resistance training; meditation practice and sharing	Summary: the 8-week mindful concurrent training journey
