# Supplementary material for: Effects of Brussels Chicory and Concurrent Exercise Training on HDL Function and Subclass Profiles in Overweight College Students: A Pilot Randomized Controlled Trial
Source: Nutrients. 2026 Aug 16;18(16):2678. doi: 10.3390/nu18162678 (PMC13516577; doi:10.3390/nu18162678)
Supplement: Supplementary file 1 [file nutrients-18-02678-s001.zip › Table S5.pdf]

Table S5. Comparison of single-intervention processes and times between Cho-MeCT and Cho-CT

Single intervention structure	Cho-MeCT		Cho-CT	
	**Content**	**Time (min)**	**Content**	**Time (min)**
Preparation phase	Mindfulness theme introduction & training overview	8	Training overview	8
	Warm-up	12	Warm-up	12
	Body awareness & interval	2	Interval	2
Main phase	Mindfulness-enhanced HIIT	10-20	HIIT	10-20
	Mindfulness theme sharing	5	Rest	5
	First group mindfulness-enhanced resistance training	15-20	First group resistance training	15-20
	Inter-set rest (exercise sensation awareness)	2	Inter-set rest	2
	Second group mindfulness-enhanced resistance training	10-15	Second group resistance training	10-15
	Recovery (exercise sensation awareness)	1	Recovery	1
Cool-down phase	Meditation relaxation & summary	10	Relaxation & summary	10

Note: Throughout the entire training process of the Cho-MeCT group, verbal guidance embedded with mindful attitudes was provided without occupying additional exercise time. Cho-CT, Brussels chicory supplementation combined with supervised concurrent resistance exercise and high-intensity interval training; Cho-MeCT, mindfulness-enhanced concurrent resistance exercise and high-intensity interval training with Brussels chicory; HIIT, high-intensity interval training.
