# Supplementary material for: Effects of Brussels Chicory and Concurrent Exercise Training on HDL Function and Subclass Profiles in Overweight College Students: A Pilot Randomized Controlled Trial
Source: Nutrients. 2026 Aug 16;18(16):2678. doi: 10.3390/nu18162678 (PMC13516577; doi:10.3390/nu18162678)
Supplement: Supplementary file 1 [file nutrients-18-02678-s001.zip › Table S6.pdf]

Table S6. Effects of different interventions on anthropometric measurements, vital capacity, HRPF, mindfulness, dietary nutrient intakes, and serum parameters*

Index	Con (n = 24)	Cho (n = 23)	Cho-CT (n = 23)	Cho-MeCT (n = 23)	G×T Effect	Cho-MeCT vs. Con		Cho-CT vs. Con		Cho vs. Con	
						MD (95% CI)	*P*	MD (95% CI)	*P*	MD (95% CI)	*P*
**Weight, kg**											
Baseline	83.48±11.52	84.78±12.43	80.204±8.26	87.10±12.81	**<0.001**						
Week 4	83.58±11.52	84.86±12.46	79.33±8.10	85.50±12.30		-1.63 (-3.11 to -0.15)	**0.023**	-1.04 (-2.06 to 0.01)	**0.045**	0.01 (-1.01 to 1.02)	1.00
Week 8	83.10±10.85	84.72±12.71	78.83±7.41	84.60±12.44		-2.04 (-3.57 to -0.51)	**0.003**	-0.62 (-1.95 to 0.70)	1.00	0.34 (-0.97 to 1.66)	1.00
**BMI, kg/m^2**											
Baseline	27.08±2.69	28.19±3.96	26.51±1.94	28.11±3.44	**0.020**						
Week 4	27.04±2.71	28.24±4.14	26.24±1.97	27.63±3.49		-0.43 (-0.93 to 0.07)	0.140	-0.24 (-0.59 to 0.12)	0.489	0.10 (-0.42 to 0.62)	1.00
Week 8	26.95±2.52	28.20±4.06	26.28±2.12	27.42±3.42		-0.54 (-1.07 to -0.02)	**0.036**	-0.10 (-0.55 to 0.35)	1.00	0.16 (-0.39 to 0.70)	1.00
**Percentage of body fat, %**											
Baseline	23.64±2.90	24.64±3.25	22.86±2.50	24.90±2.83	**<0.001**						
Week 4	23.36±2.76	24.57±3.36	21.98±2.52	23.62±2.88		-0.96 (-1.51 to -0.41)	**<0.001**	-0.62 (-1.41 to 0.17)	0.223	0.25 (-0.48 to 0.98)	1.00
Week 8	23.50±2.84	24.66±3.38	21.81±2.71	23.17±3.14		-1.55 (-2.45 to -0.66)	**<0.001**	-0.93 (-1.81 to -0.04)	**0.035**	0.20 (-0.62 to 1.01)	1.00
**Waist circumference, cm**											
Baseline	93.00±7.11	95.54±10.06	90.0±7.78	95.59±8.18	**<0.001**						
Week 4	92.36±7.34	94.41±9.93	87.71±7.62	91.95±8.74		-2.92 (-4.39 to -1.45)	**<0.001**	-1.73 (-3.65 to 0.19)	0.104	-0.42 (-2.35 to 1.51)	1.00
Week 8	92.80±7.48	95.68±10.09	87.29±7.53	90.90±8.66		-4.42 (-6.02 to -2.81)	**<0.001**	-2.59 (-5.23 to 0.05)	0.058	0.42 (-1.70 to 2.53)	1.00
**Hip circumference, cm**											
Baseline	105.54±5.04	106.48±7.17	103.35±4.45	107.44±6.13	**<0.001**						
Week 4	104.72±5.10	105.86±7.59	102.03±4.28	104.88±6.94		-1.73 (-3.21 to -0.26)	**0.012**	-0.50 (-2.00 to 1.00)	1.00	0.20 (-1.74 to 2.15)	1.00

Table S6. Continued

Index	Con (n = 24)	Cho (n = 23)	Cho-CT (n = 23)	Cho-MeCT (n = 23)	G×T Effect	Cho-MeCT vs. Con		Cho-CT vs. Con		Cho vs. Con	
						**MD (95% CI)**	***P***	**MD (95% CI)**	***P***	**MD (95% CI)**	***P***
Week 8	105.26±5.00	106.44±7.72	101.25±4.80	103.63±7.71		-3.52 (-5.84 to 1.21)	**<0.001**	-1.82 (-3.37 to -0.26)	**0.012**	0.24 (-1.46 to 1.94)	1.00
**Vital capacity, mL**											
Baseline	4932.00±809.22	4724.78±774.66	4652.83±685.11	4776.35±745.27	**<0.001**						
Week 8	5012.17±801.59	4862.3±774.80	5502.3±526.28	5466.43±683.52		593.68 (325.76 to 861.59)	**<0.001**	740.18 (423.20 to 1057.16)	**<0.001**	35.73 (-180.84 to 252.30)	1.00
**HRPF**											
Baseline	250.56±33.08	245.52±34.12	261.50±35.23	242.36±30.74	**<0.001**						
Week 8	233.66±33.45	230.30±33.12	277.13±29.53	259.62±29.49		33.65 (24.19 to 43.11)	**<0.001**	33.23 (22.54 to 43.92)	**<0.001**	1.38 (-8.00 to 10.75)	1.00
**Exercise activity level, MET-min/w**											
Baseline	1421.34±286.47	1407.24±249.62	1457.29±210.48	1446.59±206.37	0.502						
Week 4	1513.15±308.93	1499.41±209.06	1527.61±208.01	1506.24 (±224.47		-25.87 (-141.56 to 89.82)	1.00	-12.54 (-127.88 to 102.81)	1.00	-3.15 (-113.74 to 107.44)	1.00
Week 8	1558.39±180.91	1670.06±157.92	1678.53±181.05	1638.53±183.51		61.18 (-68.15 to 190.51)	1.00	93.24 (-20.01 to 206.50)	0.179	122.26 (-11.42 to 255.95)	0.095

Table S6. Continued

Index	Con (n = 24)	Cho (n = 23)	Cho-CT (n = 23)	Cho-MeCT (n = 23)	G×T Effect	Cho-MeCT vs. Con		Cho-CT vs. Con		Cho vs. Con	
						MD (95% CI)	*P*	MD (95% CI)	*P*	MD (95% CI)	*P*
**Mindfulness**											
Baseline	64.71±9.72	65.48±10.71	62.52±8.61	60.83±9.86	**<0.001**						
Week 4	65.75±9.11	66.65±10.78	63.52±7.88	67.35±8.16		4.38 (-0.78 to 9.54)	0.105	-0.66 (-6.27 to 4.95)	1.00	0.35 (05.57 to 6.27)	1.00
Week 8	65.88±9.66	65.61±10.95	64.00±11.41	75.35±11.44		12.26 (5.46 to 19.05)	**<0.001**	-0.31 (-6.94 to 6.33)	1.00	-0.82 (-6.99 to 5.35)	1.00
Week 12	66.13±9.76	66.39±9.30	63.70±6.92	73.83±10.54		10.48 (4.47 to 16.50)	**<0.001**	-0.86 (-6.21 to 4.49)	1.00	-0.29 (-5.51 to 4.93)	1.00
**Energy, kcal/d**											
Baseline	2125.47±260.26	2082.70±230.36	2196.31±207.81	2155.24±283.90	0.859						
Week 4	2082.64±233.86	2083.07±287.63	2169.37±223.02	2174.45±266.10		71.78 (-72.34 to 215.90)	1.00	39.06 (-127.94 to 206.07)	1.00	29.21 (-145.23 to 203.65)	1.00
Week 8	2118.05±210.15	2108.87±225.02	2206.67±220.31	2143.57±253.95		5.92 (-147.00 to 158.84)	1.00	40.95 (-120.03 to 201.92)	1.00	19.59 (-124.81 to164.00)	1.00
**Carbohydrate, kcal/d**											
Baseline	1195.10±180.48	1173.03±170.47	1280.57±133.09	1276.93±233.75	0.895						
Week 4	1171.07±165.77	1200.22±220.54	1279.67±180.12	1267.96±199.35		44.55 (-61.13 to 150.22)	1.00	53.92 (-79.72 to 187.57)	1.00	43.26 (-94.59 to 181.12)	1.00
Week 8	1184.80±168.59	1220.13±170.64	1284.97±128.55	1250.50±207.94		13.36 (-110.64 to 137.35)	1.00	45.49 (-64.41 to 155.38)	1.00	49.44 (-68.20 to 167.08)	1.00

Table S6. Continued

Index	Con (n = 24)	Cho (n = 23)	Cho-CT (n = 23)	Cho-MeCT (n = 23)	G×T Effect	Cho-MeCT vs. Con		Cho-CT vs. Con		Cho vs. Con	
						MD (95% CI)	*P*	MD (95% CI)	*P*	MD (95% CI)	*P*
**Protein, kcal/d**											
Baseline	326.27±30.88	323.53±33.01	325.69±31.69	302.71±36.69	0.147						
Week 4	328.18±34.82	310.81±35.13	316.73±29.20	316.35±35.69		3.72 (-21.75 to 29.19)	1.00	-11.06 (-30.42 to 8.29)	0.789	-15.56 (-40.67 to 9.55)	0.613
Week 8	332.07±41.44	313.81±32.91	323.56±39.40	313.65±27.72		-2.88 (-28.73 to 22.98)	1.00	-8.12 (-36.31 to 20.06)	1.00	-16.45 (-43.79 to 10.89)	0.674
**Fat, kcal/d**											
Baseline	604.10±59.30	586.14±50.04	590.05±55.58	575.60±50.50	0.104						
Week 4	583.39±44.68	572.04±56.48	572.97±53.28	590.14±55.78		24.77 (-11.33 to 60.87)	0.499	-1.54 (-36.41 to 33.34)	1.00	0.01 (-32.23 to 32.24)	1.00
Week 8	601.18±47.30	574.94±54.06	598.14±67.38	579.42±57.88		3.74 (-44.62 to 37.14)	1.00	5.84 (-43.56 to 55.14)	1.00	-14.89 (-48.16 to 18.38)	1.00
**Dietary fiber, kcal/d**											
Baseline	20.59±3.64	20.02±4.94	21.01±5.33	18.54±3.27	0.435						
Week 4	21.42±4.11	19.80±4.72	20.69±4.33	19.91±4.52		-0.74 (-4.01 to 2.52)	1.00	-0.89 (-4.24 to 2.47)	1.00	-1.40 (-4.61 to 1.80)	1.00
Week 8	21.07±3.00	19.40±3.27	19.80±4.27	20.76±3.97		0.45 (-2.32 to 3.23)	1.00	-1.43 (-4.30 to 1.45)	1.00	-1.46 (-4.08 to 1.16)	0.850

Table S6. Continued

Index	Con (n = 24)	Cho (n = 23)	Cho-CT (n = 23)	Cho-MeCT (n = 23)	G×T Effect	Cho-MeCT vs. Con		Cho-CT vs. Con		Cho vs. Con	
						MD (95% CI)	*P*	MD (95% CI)	*P*	MD (95% CI)	*P*
**TC, mmol/L**											
Baseline	4.35±0.44	4.55±0.46	4.47±0.62	4.45±0.46	0.056						
Week 4	4.34±0.48	4.56±0.39	4.35±0.56	4.27±0.44		-0.15 (-0.41 to 0.12)	0.892	-0.08 (-0.34 to 0.18)	1.00	0.07 (-0.17 to 0.30)	1.00
Week 8	4.34±0.53	4.47±0.37	4.23±0.41	4.18±0.41		-0.23 (-0.47 to 0.003)	**0.045**	-0.20 (-0.42 to 0.01)	0.073	-0.02 (-0.30 to 0.26)	1.00
**TG, mmol/L**											
Baseline	1.18±0.19	1.15±0.21	1.12±0.17	1.15±0.22	0.413						
Week 4	1.15±0.17	1.20±0.21	1.09±0.13	1.18±0.16		0.04 (-0.07 to 0.16)	1.00	-0.03 (-0.13 to 0.07)	1.00	0.06 (-0.08 to 0.20)	1.00
Week 8	1.20±0.15	1.19±0.15	1.11±0.12	1.14±0.14		-0.05 (-0.16 to 0.05)	1.00	-0.07 (-0.16 to 0.02)	0.187	-0.00 (-0.11 to 0.11)	1.00
**LDL-C, mmol/L**											
Baseline	2.21±0.26	2.29±0.28	2.35±0.24	2.27±0.23	0.108						
Week 4	2.26±0.24	2.20±0.25	2.22±0.23	2.24±0.20		-0.06 (-0.22 to 0.11)	1.00	-0.11 (-0.27 to 0.04)	0.299	-0.10 (-0.27 to 0.07)	0.660
Week 8	2.15±0.45	2.19±0.29	2.22±0.31	2.16±0.16		-0.02 (-0.30 to 0.26)	1.00	-0.001 (-0.33 to 0.32)	1.00	0.002 (-0.30 to 0.31)	1.00
**HDL-C, mmol/L**											
Baseline	1.23±0.27	1.29±0.33	1.24±0.28	1.17±0.24	0.245						
Week 4	1.27±0.23	1.23±0.23	1.18±0.25	1.14±0.23		-0.09 (-0.26 to 0.09)	1.00	-0.09 (-0.25 to 0.07)	0.868	-0.07 (-0.22 to 0.08)	1.00
Week 8	1.23±0.25	1.28±0.17	1.30±0.25	1.21±0.16		0.02 (-0.10 to 0.14)	1.00	0.07 (-0.07 to 0.22)	1.00	0.02 (-0.14 to 0.18)	1.00

Table S6. Continued

Index	Con (n = 24)	Cho (n = 23)	Cho-CT (n = 23)	Cho-MeCT (n = 23)	G×T Effect	Cho-MeCT vs. Con		Cho-CT vs. Con		Cho vs. Con	
						MD (95% CI)	***P***	MD (95% CI)	***P***	MD (95% CI)	***P***
**Apo A-I, mg/L**											
Baseline	1119.40±245.51	1161.97±297.00	1173.71±246.59	1071.71±229.48	0.352						
Week 4	1145.76±264.27	1096.92±248.66	1107.83±207.29	1078.19±207.29		-36.55 (-210.83 to 137.73)	1.00	-73.26 (-232.51 to 85.99)	1.00	-76.34 (-234.26 to 81.58)	1.00
Week 8	1154.89±295.06	1230.30±185.63	1308.29±300.86	1184.22±229.03		60.36 (-86.74 to 207.45)	1.00	118.06 (-69.36 to 305.47)	0.579	47.91 (-139.88 to 235.69)	1.00
**AST, U/L**											
Baseline	24.67±5.94	21.41±7.00	23.19±7.39	21.38±8.08	0.153						
Week 4	22.23±7.75	23.80±8.78	24.53±9.75	24.46±7.20		3.41 (-1.61 to 9.43)	0.808	2.83 (-4.20 to 9.85)	1.00	2.74 (-3.51 to 9.00)	1.00
Week 8	26.74±6.56	23.53±9.22	22.12±7.43	25.58±7.01		0.04 (-5.36 to 5.43)	1.00	-4.08 (-8.96 to 0.80)	0.165	-2.03 (-8.21 to 4.15)	1.00
**ALT, U/L**											
Baseline	22.19±5.10	20.39±5.51	19.44±8.21	19.79±6.47	0.063						
Week 4	19.34±6.74	22.99±4.27	20.84±7.83	19.97±6.34		1.46 (-3.61 to 6.53)	1.00	2.45 (-3.11 to 8.01)	1.00	4.27 (-0.04 to 8.57)	0.053
Week 8	19.95±5.88	21.01±6.04	22.41±7.65	22.04±5.49		2.91 (-1.84 to 7.67)	0.634	3.40 (-2.08 to 8.89)	0.611	1.68 (-3.24 to 6.60)	1.00
**Alb, g/L**											
Baseline	38.72±3.84	40.68±3.78	37.68±4.77	39.25±3.35	0.205						
Week 4	39.21±3.19	38.80±3.61	37.86±4.83	37.53±4.62		-1.95 (-4.95 to 1.05)	0.521	-0.82 (-3.80 to 2.16)	1.00	-1.41 (-4.16 to 1.35)	1.00
Week 8	39.36±4.11	38.74±4.13	39.18±4.02	38.28±5.22		-1.35 (-4.45 to 1.76)	1.00	0.35 (-2.75 to 3.45)	1.00	-1.62 (-4.45 to 1.20)	0.779

Table S6. Continued

Index	Con (n = 24)	Cho (n = 23)	Cho-CT (n = 23)	Cho-MeCT (n = 23)	G×T Effect	Cho-MeCT vs. Con		Cho-CT vs. Con		Cho vs. Con	
						MD (95% CI)	*P*	MD (95% CI)	*P*	MD (95% CI)	*P*
**Cr, µmol/L**											
Baseline	77.42±16.82	76.20±15.39	81.88±11.52	74.37±16.47	0.362						
Week 4	80.55±14.11	74.34±14.37	74.51±19.13	81.81±11.97		2.21 (-7.90 to 12.33)	1.00	-7.43 (-21.00 to 6.15)	1.00	-5.83 (-16.81 to 5.15)	0.966
Week 8	77.67±13.86	75.75±12.68	80.65±12.96	82.90±10.58		6.17 (-4.29 to 16.64)	0.717	1.59 (-8.48 to 11.66)	1.00	-1.55 (-12.00 to 8.91)	1.00
**BUN, mmol/L**											
Baseline	4.35±0.61	4.48±0.69	4.23±0.60	4.41±0.62	0.231						
Week 4	4.19±0.48	4.20±0.47	4.45±0.46	4.35±0.60		0.04 (-0.40 to 0.48)	1.00	0.20 (-0.19 to 0.58)	1.00	-0.13 (-0.52 to 0.27)	1.00
Week 8	4.48±0.63	4.38±0.67	4.11±0.64	4.52±0.72		0.03 (-0.50 to 0.55)	1.00	-0.33 (-0.80 to 0.15)	0.421	-0.14 (-0.65 to 0.37)	1.00
**PCA, nmol/L**											
Baseline	5.87±1.72	5.44±2.14	6.11±2.44	5.94±2.16	**<0.001**						
Week 4	7.26±2.12	10.31±2.99	10.59±3.16	12.14±2.84		4.83 (2.87 to 6.79)	**<0.001**	3.13 (1.08 to 5.18)	**<0.001**	3.40 (1.48 to 5.32)	**<0.001**
Week 8	6.94±1.77	14.13±4.72	13.11±4.36	13.58±3.52		6.58 (4.76 to 8.41)	**<0.001**	5.97 (3.76 to 8.17)	**<0.001**	7.54 (5.35 to 9.74)	**<0.001**

* Data are mean ± SD. Analyses were performed using generalized estimating equations (GEE). For all outcomes presented in this table, covariates included baseline measurement values, time, group, and group-by-time interaction. Pairwise comparisons were performed using linear contrasts with Bonferroni correction. Boldfaced *P* values denote significance after correction.

Alb, Albumin; ALT, Alanine transaminase; Apo A-I, Apolipoprotein A-I; AST, Aspartate transferase; BMI, body mass index; Cho, Brussels chicory; BUN, Blood urea nitrogen; Cr, Creatinine; Cho-CT, Brussels chicory supplementation combined with supervised concurrent resistance exercise and high-intensity interval training; Cho-MeCT, mindfulness-enhanced concurrent resistance exercise and high-intensity interval training with Brussels chicory; Con, control; HDL-C, high-density lipoprotein cholesterol; LDL-C, low-density lipoprotein cholesterol; HRPF, Health-related physical fitness; MET-min/w, metabolic equivalent of task-minutes per week; PCA, protocatechuic acid; TC, total cholesterol; TG, triglyceride; G×T, group-by-time interaction effect; MD, mean difference; CI, confidence interval.
