# Supplementary material for: Effects of Brussels Chicory and Concurrent Exercise Training on HDL Function and Subclass Profiles in Overweight College Students: A Pilot Randomized Controlled Trial
Source: Nutrients. 2026 Aug 16;18(16):2678. doi: 10.3390/nu18162678 (PMC13516577; doi:10.3390/nu18162678)
Supplement: Supplementary file 1 [file nutrients-18-02678-s001.zip › Table S7.pdf]

Table S7. Comparisons of Cho-MeCT, Cho-CT, and Cho on anthropometric measurements, vital capacity, HRPF, mindfulness, dietary nutrient intakes, and serum parameters*

Index	Cho (n = 23)	Cho-CT (n = 23)	Cho-MeCT (n = 23)	G×T Effect	Cho-MeCT vs. Cho-CT		Cho-CT vs. Cho		Cho-MeCT vs. Cho	
					MD (95% CI)	*P*	MD (95% CI)	*P*	MD (95% CI)	*P*
**Weight, kg**										
Baseline	84.78±12.43	80.204±8.26	87.10±12.81	**<0.001**						
Week 4	84.86±12.46	79.33±8.10	85.50±12.30		-0.59 (-2.06 to 0.88)	1.00	-1.04 (-2.04 to 0.05)	**0.035**	-1.63 (-3.12 to 0.15)	**0.022**
Week 8	84.72±12.71	78.83±7.41	84.60±12.44		-1.42 (-2.90 to 0.05)	0.066	-0.97 (-2.21 to 0.28)	0.246	-2.39 (-3.87 to -0.90)	**<0.001**
**BMI, kg/m^2**										
Baseline	28.19±3.96	26.51±1.94	28.11±3.44	**0.020**						
Week 4	28.24±4.14	26.24±1.97	27.63±3.49		-0.19 (-0.68 to 0.30)	1.00	-0.33 (-0.84 to 0.18)	0.519	-0.52 (-1.14 to 0.10)	0.155
Week 8	28.20±4.06	26.28±2.12	27.42±3.42		-0.45 (-0.95 to 0.05)	0.110	-0.26 (-0.78 to 0.27)	1.00	-0.70 (-1.29 to 0.11)	**0.011**
**Percentage of body fat, %**										
Baseline	24.64±3.25	22.86±2.50	24.90±2.83	**<0.001**						
Week 4	24.57±3.36	21.98±2.52	23.62±2.88		-0.34 (-1.22 to 0.54)	1.00	-0.87 (-1.87 to 0.13)	0.129	-1.21 (-2.01 to -0.41)	**<0.001**
Week 8	24.66±3.38	21.81±2.71	23.17±3.14		-0.63 (-1.62 to 0.37)	0.574	-1.12 (-2.05 to -0.20)	**0.008**	-1.75 (-2.68 to -0.82)	**<0.001**
**Waist circumference, cm**										
Baseline	95.54±10.06	90.0±7.78	95.59±8.18	**<0.001**						
Week 4	94.41±9.93	87.71±7.62	91.95±8.74		-1.19 (-3.26 to 0.87)	0.766	-1.31 (-3.72 to 1.10)	0.911	-2.50 (-4.47 to -0.52)	**0.005**
Week 8	95.68±10.09	87.29±7.53	90.90±8.66		-1.83 (-4.54 to 0.88)	0.453	-3.00 (-6.05 to 0.05)	0.056	-4.83 (-6.95 to -2.71)	**<0.001**

Table S7. Continued

Index	Cho (n = 23)	Cho-CT (n = 23)	Cho-MeCT (n = 23)	G×T Effect	Cho-MeCT vs. Cho-CT		Cho-CT vs. Cho		Cho-MeCT vs. Cho	
					MD (95% CI)	*P*	MD (95% CI)	*P*	MD (95% CI)	*P*
**Hip circumference, cm**										
Baseline	106.48±7.17	103.35±4.45	107.44±6.13	**<0.001**						
Week 4	105.86±7.59	102.03±4.28	104.88±6.94		-1.23 (-2.89 to 0.43)	0.304	-0.70 (-2.77 to 1.36)	1.00	-1.94 (-4.03 to 0.16)	0.089
Week 8	106.44±7.72	101.25±4.80	103.63±7.71		-1.71 (-4.05 to 0.64)	0.327	-2.06 (-3.76 to -0.35)	**0.009**	-3.76 (-6.21 to -1.32)	**<0.001**
**Vital capacity, mL**										
Baseline	4724.78±774.66	4652.83±685.11	4776.35±745.27	**<0.001**						
Week 8	4862.3±774.80	5502.3±526.28	5466.43±683.52		-146.50 (-493.07 to 200.07)	1.00	704.45 (395.56 to 1013.34)	**<0.001**	557.95 (309.49 to 806.40)	**<0.001**
**HRPF**										
Baseline	245.52±34.12	261.50±35.23	242.36±30.74	**<0.001**						
Week 8	230.30±33.12	277.13±29.53	259.62±29.49		0.42 (-8.38 to 9.22)	1.00	31.85 (23.11 to 40.60)	**<0.001**	32.27 (25.30 to 39.25)	**<0.001**
**Exercise activity level, MET-min/w**										
Baseline	1407.24±249.62	1457.29±210.48	1446.59±206.37	0.502						
Week 4	1499.41±209.06	1527.61±208.01	1506.24±224.47		-13.33 (-106.43 to 79.77)	1.00	-9.38 (-96.84 to 78.07)	1.00	-22.72 (-110.67 to 65.24)	1.00
Week 8	1670.06±157.92	1678.53±181.05	1638.53±183.51		-32.06 (-147.53 to 83.41)	1.00	-29.02 (-148.91 to 90.87)	1.00	-61.08 (-196.20.56 to 74.03)	1.00

Table S7. Continued

Index	Cho (n = 23)	Cho-CT (n = 23)	Cho-MeCT (n = 23)	G×T Effect	Cho-MeCT vs. Cho-CT		Cho-CT vs. Cho		Cho-MeCT vs. Cho	
					**MD (95% CI)**	***P***	**MD (95% CI)**	***P***	**MD (95% CI)**	***P***
**Mindfulness**										
Baseline	65.48±10.71	62.52±8.61	60.83±9.86	**<0.001**						
Week 4	66.65±10.78	63.52±7.88	67.35±8.16		5.04 (-0.56 to 10.64)	0.105	-1.01 (-7.26 to 5.24)	1.00	4.03 (-1.83 to 9.89)	0.416
Week 8	65.61±10.95	64.00±11.41	75.35±11.44		12.56 (4.50 to 20.62)	**<0.001**	0.51 (-6.91 to 7.93)	1.00	13.07 (5.53 to 20.62)	**<0.001**
Week 12	66.39±9.30	63.70±6.92	73.83±10.54		11.35 (5.15 to 17.55)	**<0.001**	-0.58 (-5.92 to 4.77)	1.00	10.77 (4.76 to 16.78)	**<0.001**
**Energy, kcal/d**										
Baseline	2082.70±230.36	2196.31±207.81	2155.24±283.90	0.859						
Week 4	2083.07±287.63	2169.37±223.02	2174.45±266.10		32.72 (-129.95 to 195.38)	1.00	9.86 (-178.72 to 198.44)	1.00	42.57 (-125.17 to 210.32)	1.00
Week 8	2108.87±225.02	2206.67±220.31	2143.57±253.95		-35.02 (-203.79 to 133.75)	1.00	21.35 (-137.49 to 100.20)	1.00	-13.67 (-163.21 to 135.87)	1.00
**Carbohydrate, kcal/d**										
Baseline	1173.03±170.47	1280.57±133.09	1276.93±233.75	0.895						
Week 4	1200.22±220.54	1279.67±180.12	1267.96±199.35		-9.38 (-139.78 to 121.02)	1.00	10.66 (-145.29 to 166.62)	1.00	1.28 (-131.56 to 134.13)	1.00

Table S7. Continued

Index	Cho (n = 23)	Cho-CT (n = 23)	Cho-MeCT (n = 23)	G×T Effect	Cho-MeCT vs. Cho-CT		Cho-CT vs. Cho		Cho-MeCT vs. Cho	
					MD (95% CI)	***P***	MD (95% CI)	***P***	MD (95% CI)	***P***
Week 8	1220.13±170.64	1284.97±128.55	1250.50±207.94		-32.13 (-153.76 to 89.50)	1.00	-3.95 (-118.96 to 111.06)	1.00	-36.08 (-164.47 to 92.30)	1.00
**Protein, kcal/d**										
Baseline	323.53±33.01	325.69±31.69	302.71±36.69	0.147						
Week 4	310.81±35.13	316.73±29.20	316.35±35.69		14.78 (-5.95 to 35.51)	0.359	4.50 (-16.15 to 25.14)	1.00	19.28 (-7.04 to 45.59)	0.320
Week 8	313.81±32.91	323.56±39.40	313.65±27.72		5.24 (-18.32 to 28.81)	1.00	8.33 (-17.67 to 34.33)	1.00	13.57 (-9.68 to 36.82)	0.741
**Fat, kcal/d**										
Baseline	586.14±50.04	590.05±55.58	575.60±50.50	0.104						
Week 4	572.04±56.48	572.97±53.28	590.14±55.78		26.30 (-13.48 to 66.09)	0.487	-1.54 (-38.41 to 35.33)	1.00	24.76 (-12.95 to 62.47)	0.499
Week 8	574.94±54.06	598.14±67.38	579.42±57.88		-9.58 (-56.00 to 36.84)	1.00	20.73 (-19.86 to 61.32)	1.00	11.15 (-17.82 to 40.12)	1.00
**Dietary fiber, kcal/d**										
Baseline	20.02±4.94	21.01±5.33	18.54±3.27	0.435						
Week 4	19.80±4.72	20.69±4.33	19.91±4.52		0.15 (-3.70 to 4.00)	1.00	0.51 (-3.26 to 4.28)	1.00	0.66 (-3.02 to 4.34)	1.00
Week 8	19.40±3.27	19.80±4.27	20.76±3.97		1.88 (01.34 to 5.10)	0.741	0.04 (-3.02 to 3.09)	1.00	1.91 (-1.07 to 4.90)	0.546

Table S7. Continued

Index	Cho (n = 23)	Cho-CT (n = 23)	Cho-MeCT (n = 23)	G×T Effect	Cho-MeCT vs. Cho-CT		Cho-CT vs. Cho		Cho-MeCT vs. Cho	
					MD (95% CI)	*P*	MD (95% CI)	*P*	MD (95% CI)	*P*
**TC, mmol/L**										
Baseline	4.55±0.46	4.47±0.62	4.45±0.46	0.056						
Week 4	4.56±0.39	4.35±0.56	4.27±0.44		-0.06 (-0.33 to 0.20)	1.00	-0.15 (-0.39 to 0.09)	0.587	-0.21 (-0.46 to 0.03)	0.140
Week 8	4.47±0.37	4.23±0.41	4.18±0.41		-0.03 (-0.23 to 0.17)	1.00	-0.18 (-0.44 to 0.07)	0.361	-0.21 (-0.48 to 0.06)	0.237
**TG, mmol/L**										
Baseline	1.15±0.21	1.12±0.17	1.15±0.22	0.413						
Week 4	1.20±0.21	1.09±0.13	1.18±0.16		0.07 (-0.04 to 0.19)	0.497	-0.09 (-0.23 to 0.05)	0.525	-0.02 (-0.17 to 0.13)	1.00
Week 8	1.19±0.15	1.11±0.12	1.14±0.14		0.02 (-0.10 to 0.14)	1.00	-0.07 (-0.19 to 0.05)	0.751	-0.05 (-0.19 to 0.09)	1.00
**LDL-C, mmol/L**										
Baseline	2.29±0.28	2.35±0.24	2.27±0.23	0.108						
Week 4	2.20±0.25	2.22±0.23	2.24±0.20		0.06 (-0.08 to 0.20)	1.00	-0.01 (-0.16 to 0.14)	1.00	0.05 (-0.11 to 0.21)	1.00
Week 8	2.19±0.29	2.22±0.31	2.16±0.16		-0.02 (-0.22 to 0.18)	1.00	-0.003 (-0.23 to 0.22)	1.00	-0.02 (-0.19 to 0.15)	1.00
**HDL-C, mmol/L**										
Baseline	1.29±0.33	1.24±0.28	1.17±0.24	0.245						
Week 4	1.23±0.23	1.18±0.25	1.14±0.23		0.003 (-0.17 to 0.17)	1.00	-0.02 (-0.17 to 0.13)	1.00	-0.02 (-0.18 to 0.15)	1.00
Week 8	1.28±0.17	1.30±0.25	1.21±0.16		-0.05 (-0.18 to 0.09)	1.00	0.05 (-0.12 to 0.22)	1.00	0.01 (-0.15 to 0.16)	1.00

Table S7. Continued

Index	Cho (n = 23)	Cho-CT (n = 23)	Cho-MeCT (n = 23)	G×T Effect	Cho-MeCT vs. Cho-CT		Cho-CT vs. Cho		Cho-MeCT vs. Cho	
					MD (95% CI)	*P*	MD (95% CI)	*P*	MD (95% CI)	*P*
**Apo A-I, mg/L**										
Baseline	1161.97±297.00	1173.71±246.59	1071.71±229.48	0.352						
Week 4	1096.92±248.66	1107.83±207.29	1078.19±207.29		36.71 (-125.85 to 199.27)	1.00	3.08 (-141.53 to 147.69)	1.00	39.79 (-121.31 to 200.89)	1.00
Week 8	1230.30±185.63	1308.29±300.86	1184.22±229.03		-57.70 (-230.62 to 115.22)	1.00	70.15 (-139.40 to 279.70)	1.00	12.45 (-161.20 to 186.11)	1.00
**AST, U/L**										
Baseline	21.41±7.00	23.19±7.39	21.38±8.08	0.153						
Week 4	23.80±8.78	24.53±9.75	24.46±7.20		0.58 (-6.37 to 7.54)	1.00	0.08 (-7.08 to 7.25)	1.00	0.67 (-5.56 to 6.90)	1.00
Week 8	23.53±9.22	22.12±7.43	25.58±7.01		4.12 (-1.63 to 9.86)	0.352	-2.05 (-8.62 to 4.51)	1.00	2.06 (-4.79 to 8.91)	1.00
**ALT, U/L**										
Baseline	20.39±5.51	19.44±8.21	19.79±6.47	0.063						
Week 4	22.99±4.27	20.84±7.83	19.97±6.34		-0.99 (-6.49 to 4.50)	1.00	-1.81 (-6.67 to 3.04)	1.00	-2.81 (-7.13 to 1.51)	0.519
Week 8	21.01±6.04	22.41±7.65	22.04±5.49		-0.49 (-5.82 to 4.84)	1.00	1.72 (-3.79 to 7.24)	1.00	1.23 (-3.56 to 6.03)	1.00
**Alb, g/L**										
Baseline	40.68±3.78	37.68±4.77	39.25±3.35	0.205						
Week 4	38.80±3.61	37.86±4.83	37.53±4.62		-1.13 (-4.70 to 2.43)	1.00	0.59 (-2.69 to 3.87)	1.00	-0.54 (-3.80 to 2.71)	1.00
Week 8	38.74±4.13	39.18±4.02	38.28±5.22		-1.70 (-5.42 to 2.02)	1.00	1.97 (-1.45 to 5.40)	1.00	0.28 (-3.15 to 3.71)	1.00

Table S7. Continued

Index	Cho (n = 23)	Cho-CT (n = 23)	Cho-MeCT (n = 23)	G×T Effect	Cho-MeCT vs. Cho-CT		Cho-CT vs. Cho		Cho-MeCT vs. Cho	
					MD (95% CI)	*P*	MD (95% CI)	*P*	MD (95% CI)	*P*
**Cr, µmol/L**										
Baseline	76.20±15.39	81.88±11.52	74.37±16.47	0.362						
Week 4	74.34±14.37	74.51±19.13	81.81±11.97		9.64 (-3.50 to 22.78)	1.00	-1.59 (-15.44 to 12.26)	1.00	8.05 (-2.12 to 18.22)	0.221
Week 8	75.75±12.68	80.65±12.96	82.90±10.58		4.58 (-5.71 to 14.88)	1.00	3.14 (-7.09 to 13.36)	1.00	7.72 (-2.89 to 18.33)	0.330
**BUN, mmol/L**										
Baseline	4.48±0.69	4.23±0.60	4.41±0.62	0.231						
Week 4	4.20±0.47	4.45±0.46	4.35±0.60		-0.16 (-0.59 to 0.28)	1.00	0.32 (-0.08 to 0.72)	0.196	0.17 (-0.28 to 0.61)	1.00
Week 8	4.38±0.67	4.11±0.64	4.52±0.72		0.35 (-0.19 to 0.90)	0.531	-0.19 (-0.71 to 0.33)	1.00	0.16 (-0.40 to 0.73)	1.00
**PCA, nmol/L**										
Baseline	5.44±2.14	6.11±2.44	5.94±2.16	**<0.001**						
Week 4	10.31±2.99	10.59±3.16	12.14±2.84		1.70 (-0.09 to 3.49)	0.074	-0.27 (-2.01 to 1.46)	1.00	1.43 (-0.20 to 3.05)	0.124
Week 8	14.13±4.72	13.11±4.36	13.58±3.52		0.62 (-2.23 to 3.46)	1.00	-1.58 (-4.66 to 1.51)	1.00	-0.96 (-3.79 to 1.87)	1.00

* Data are mean ± SD. Analyses were performed using generalized estimating equations (GEE). For all outcomes presented in this table, covariates included baseline measurement values, time, group, and group-by-time interaction. Pairwise comparisons were performed using linear contrasts with Bonferroni correction. Boldfaced P values denote significance after correction.

Alb, Albumin; ALT, Alanine transaminase; Apo A-I, Apolipoprotein A-I; AST, Aspartate transferase; BMI, body mass index; Cho, Brussels chicory; BUN, Blood urea nitrogen; Cr, Creatinine; Cho-CT, Brussels chicory supplementation combined with supervised concurrent resistance exercise and high-intensity interval training; Cho-MeCT, mindfulness-enhanced concurrent resistance exercise and high-intensity interval training with Brussels chicory; Con, control; HDL-C, high-density lipoprotein cholesterol; LDL-C, low-density lipoprotein cholesterol; HRPF, Health-related physical fitness; MET-min/w, metabolic equivalent of task-minutes per week; PCA, protocatechuic acid; TC, total cholesterol; TG, triglyceride; G×T, group-by-time interaction effect; MD, mean difference; CI, confidence interval.
