# Supplementary material for: Effects of Brussels Chicory and Concurrent Exercise Training on HDL Function and Subclass Profiles in Overweight College Students: A Pilot Randomized Controlled Trial
Source: Nutrients. 2026 Aug 16;18(16):2678. doi: 10.3390/nu18162678 (PMC13516577; doi:10.3390/nu18162678)
Supplement: Supplementary file 1 [file nutrients-18-02678-s001.zip › Table S8.pdf]

Table S8. Effects of different interventions on primary and secondary outcomes*

Index	Con (n = 24)	Cho (n = 23)	Cho-CT (n = 23)	Cho-MeCT (n = 23)	G×T Effect	Cho-MeCT vs.Con		Cho-CT vs.Con		Cho vs.Con	
						MD (95% CI)	*P*	MD (95% CI)	*P*	MD (95% CI)	*P*
**HDL-CEC, %**											
Baseline	8.13±1.73	7.85±2.20	8.08±1.76	8.06±1.39	**<0.001**						
Week 4	8.08±1.59	8.25±2.09	8.70±2.01	8.68±1.89		0.63 (-0.61 to 1.87)	1.00	0.66 (-0.60 to 1.93)	1.00	0.36 (-0.87 to 1.59)	1.00
Week 8	7.93±1.49	8.93±1.36	9.67±2.01	9.42±1.28		1.52 (0.81 to 2.23)	**<0.001**	1.78 (0.77 to 2.80)	**<0.001**	1.18 (0.40 to 1.97)	**<0.001**
**Pre-*β*-HDL, mmol/L**											
Baseline	112.44±32.20	120.69±33.68	115.19±28.28	107.95±22.69	**<0.001**						
Week 4	118.23±30.20	112.65±21.18	117.95±32.88	107.74±38.90		-8.34 (-34.37 to 17.68)	1.00	-1.60 (-23.83 to 20.63)	1.00	-9.52 (-29.99 to 10.95)	1.00
Week 8	122.15±27.03	62.46±15.80	74.07±15.80	64.03±22.83		-55.98 (-76.31 to -35.66)	**<0.001**	-49.40 (-69.31 to -29.48)	**<0.001**	-63.63 (-84.94 to -42.32)	**<0.001**
**Pre-*β₁*-HDL, mg/L**											
Baseline	66.09±18.85	69.12±22.20	67.51±17.56	64.09±15.29	**<0.001**						
Week 4	69.45±17.49	66.48±17.74	70.05±19.73	62.72±20.53		-5.74 (-20.34 to 8.85)	1.00	-0.09 (-13.94 to 13.76)	1.00	-4.44 (-17.12 to 8.25)	1.00
Week 8	68.61±21.54	34.37±14.10	40.57±10.67	34.07±13.85		-33.57 (-47.24 to -19.89)	**<0.001**	-28.74 (-42.44 to -15.03)	**<0.001**	-35.72 (-50.55 to -20.88)	**<0.001**
**Pre-*β₂*-HDL, mg/L**											
Baseline	46.35±14.34	51.57±19.13	47.68±14.79	43.86±11.28	**<0.001**						
Week 4	48.78±14.63	46.16±12.98	47.90±15.50	45.02±19.81		-2.63 (-15.86 to 10.61)	**1.00**	-1.50 (-12.09 to 9.10)	**1.00**	-5.01 (-15.36 to 5.35)	**1.00**

Table S8. Continued

Index	Con (n = 24)	Cho (n = 23)	Cho-CT (n = 23)	Cho-MeCT (n = 23)	G×T Effect	Cho-MeCT vs.Con MD (95% CI)	Cho-MeCT vs.Con *P*	Cho-CT vs.Con MD (95% CI)	Cho-CT vs.Con *P*	Cho vs.Con MD (95% CI)	Cho vs.Con *P*
Week 8	53.54±10.66	28.09±12.81	33.51±9.22	29.96±13.16		-22.45 (-32.30 to -12.60)	**<0.001**	-20.65 (-29.67 to -11.62)	**<0.001**	-27.84 (-38.42 to -17.26)	**<0.001**
**HDL_3, mg/L**											
Baseline	550.72±113.58	576.86±134.88	573.03±140.83	538.40±115.86	**<0.001**						
Week 4	556.48±161.50	530.96±154.58	546.50±111.66	513.20±91.77		-36.44 (-130.49 to 57.61)	1.00	-22.35 (-115.59 to 70.90)	1.00	-40.02 (-150.34 to 70.31)	1.00
Week 8	561.51±119.43	442.17±88.30	453.20±93.03	415.61±121.63		-139.07 (-217.59 to -60.54)	**<0.001**	-120.68 (-196.85 to -44.52)	**<0.001**	-133.84 (-214.06 to -53.62)	**<0.001**
**HDL_{3c}, mg/L**											
Baseline	79.04±20.30	81.79±22.16	85.29±24.62	84.30±19.06	0.958						
Week 4	76.04±22.34	75.25±19.34	78.84±24.43	80.68±16.57		1.50 (-12.44 to 15.45)	1.00	-0.93 (-14.77 to 12.91)	1.00	-2.43 (-15.08 to 10.22)	1.00
Week 8	82.60±20.10	86.05±18.40	90.18±24.96	93.58±19.81		7.84 (-5.92 to 21.60)	0.796	3.85 (-13.32 to 21.02)	1.00	1.81 (-13.71 to 17.32)	1.00
**HDL_{3b}, mg/L**											
Baseline	241.02±60.51	249.44±65.63	249.52±75.24	232.57±56.82	**<0.001**						
Week 4	250.31±93.99	247.20±94.97	251.56±80.35	221.32±62.46		-24.11 (-83.39 to 35.16)	1.00	-3.65 (-64.48 to 57.18)	1.00	-7.96 (-78.20 to 62.29)	1.00

Table S8. Continued

Index	Con (n = 24)	Cho (n = 23)	Cho-CT (n = 23)	Cho-MeCT (n = 23)	G×T Effect	Cho-MeCT vs.Con MD (95% CI)	Cho-MeCT vs.Con *P*	Cho-CT vs.Con MD (95% CI)	Cho-CT vs.Con *P*	Cho vs.Con MD (95% CI)	Cho vs.Con *P*
Week 8	249.32±74.33	157.46±52.60	172.74±81.91	148.84±72.68		-95.60 (-146.85 to -44.36)	**<0.001**	-81.48 (-133.70 to -29.26)	**<0.001**	-96.71 (-147.88 to -45.54)	**<0.001**
**HDL_{3a}, mg/L**											
Baseline	230.67±65.40	245.63±92.34	238.21±70.55	221.53±62.95	**0.030**						
Week 4	230.13±58.72	208.51±56.66	216.11±69.13	211.20±51.29		-14.46 (-56.32 to 27.41)	1.00	-17.72 (-63.65 to 28.21)	1.00	-28.95 (-69.87 to 11.98)	0.372
Week 8	229.60±58.01	198.66±34.20	190.28±48.43	173.19±39.14		-51.94 (-84.34 to -19.53)	**<0.001**	-43.00 (-79.83 to -6.17)	**0.012**	-38.26 (-79.40 to 2.88)	0.085
**HDL_2, mg/L**											
Baseline	456.24±125.92	464.12±292.97	485.50±216.74	425.36±189.88	**<0.001**						
Week 4	471.06±131.09	453.31±157.96	443.39±170.93	457.24±108.41		-0.33 (-95.74 to 95.07)	1.00	-40.44 (-137.21 to 56.32)	1.00	-21.18 (-109.83 to 67.47)	1.00
Week 8	471.23±181.58	725.66±139.03	781.01±311.77	704.58±129.20		246.84 (130.81 to 362.87)	**<0.001**	297.01 (98.16 to 495.86)	**<0.001**	251.00 (102.07 to 399.03)	**<0.001**
**HDL_{2a}, mg/L**											
Baseline	259.86±76.80	264.38±81.64	265.84±76.13	242.46±80.89	**0.040**						
Week 4	268.71±65.91	278.51±82.42	271.38±71.82	256.48±57.67		-2.78 (-57.21 to 51.66)	1.00	-0.57 (-53.66 to 52.52)	1.00	7.35 (-48.03 to 62.74)	1.00

Table S8. Continued

Index	Con (n = 24)	Cho (n = 23)	Cho-CT (n = 23)	Cho-MeCT (n = 23)	G×T Effect	Cho-MeCT vs.Con		Cho-CT vs.Con		Cho vs.Con	
						MD (95% CI)	*P*	MD (95% CI)	*P*	MD (95% CI)	*P*
Week 8	257.11±80.03	319.21±76.09	329.37±95.61	286.03±82.49		38.36 (-8.82 to 85.55)	0.192	69.01 (13.00 to 125.02)	**0.007**	59.64 (0.40 to 118.88)	**0.047**
**HDL_{2b}, mg/L**											
Baseline	196.38±77.23	199.74±242.01	219.65±179.57	182.91±150.71	**<0.001**						
Week 4	202.35±103.34	174.80±125.32	172.01±126.07	200.76±107.29		3.34 (-76.22 to 82.90)	1.00	-38.87 (-117.96 to 40.22)	1.00	-28.78 (-110.94 to 53.38)	1.00
Week 8	214.11±122.25	406.46±93.68	451.64±237.04	418.55±96.12		209.38 (119.30 to 299.46)	**<0.001**	229.00 (74.25 to 383.76)	**0.001**	191.12 (86.86 to 295.37)	**<0.001**
**LCAT, nmol · L^{-1} · h^{-1}**											
Baseline	113.81±17.44	126.27±14.25	123.12±17.84	121.90±16.26	0.350						
Week 4	126.91±15.27	131.44±17.27	124.87±21.69	129.85±22.02		-0.07 (-14.47 to 14.34)	1.00	-5.51 (-19.25 to 8.23)	1.00	-0.11 (-13.14 to 12.93)	1.00
Week 8	128.35±13.82	141.75±17.82	129.02±12.38	136.57±16.82		5.21 (-7.81 to 18.23)	1.00	-2.79 (-14.23 to 8.64)	1.00	8.76 (-4.54 to 22.05)	0.493
**CETP, nmol · L^{-1} · h^{-1}**											
Baseline	87.84±7.91	86.36±8.12	85.34±7.80	83.64±9.31	0.536						
Week 4	85.91±5.01	89.08±7.49	88.78±9.19	84.93±8.01		0.78 (-4.88 to 6.44)	1.00	3.92 (-2.28 to 10.13)	0.572	3.80 (-1.73 to 9.32)	0.419

Table S8. Continued

Index	Con (n = 24)	Cho (n = 23)	Cho-CT (n = 23)	Cho-MeCT (n = 23)	G×T Effect	Cho-MeCT vs.Con MD (95% CI)	Cho-MeCT vs.Con *P*	Cho-CT vs.Con MD (95% CI)	Cho-CT vs.Con *P*	Cho vs.Con MD (95% CI)	Cho vs.Con *P*
Week 8	90.90±11.06	91.86±9.78	90.55±11.46	86.01±9.58		-3.12 (-10.81 to 4.56)	1.00	0.70 (-8.13 to 9.53)	1.00	1.59 (-6.14 to 9.31)	1.00

* Data are mean ± SD. Analyses were performed using generalized estimating equations (GEE). For the primary outcome (HDL-CEC), covariates included baseline measurement values, time, group, group-by-time interaction, and baseline body mass index (BMI). For the other outcomes, covariates included baseline measurement values, time, group, and group-by-time interaction (without baseline BMI adjustment). Pairwise comparisons were performed using linear contrasts with Bonferroni correction. Boldfaced P values denote significance after correction.

CETP, Cholesteryl ester transfer protein; Cho-CT, Brussels chicory supplementation combined with supervised concurrent resistance exercise and high-intensity interval training; Cho-MeCT, mindfulness-enhanced concurrent resistance exercise and high-intensity interval training with Brussels chicory; Con, control; HDL-C, high-density lipoprotein cholesterol; HDL-CEC, high-density lipoprotein cholesterol efflux capacity; LCAT, Lecithin: cholesterol acyltransferase; G×T, group-by-time interaction effect; MD, mean difference; CI, confidence interval.
