# Supplementary material for: Effects of Brussels Chicory and Concurrent Exercise Training on HDL Function and Subclass Profiles in Overweight College Students: A Pilot Randomized Controlled Trial
Source: Nutrients. 2026 Aug 16;18(16):2678. doi: 10.3390/nu18162678 (PMC13516577; doi:10.3390/nu18162678)
Supplement: Supplementary file 1 [file nutrients-18-02678-s001.zip › Table S9.pdf]

Table S9. Comparisons of Cho, Cho-CT, and Cho-MeCT on primary and secondary outcomes *

Index	Cho (n = 23)	Cho-CT (n = 23)	Cho-MeCT (n = 23)	G×T Effect	Cho-MeCT vs.Cho-CT		Cho-CT vs.Cho		Cho-MeCT vs.Cho	
					MD (95% CI)	*P*	MD (95% CI)	*P*	MD (95% CI)	*P*
**HDL-CEC, %**										
Baseline	7.85±2.20	8.08±1.76	8.06±1.39	**<0.001**						
Week 4	8.25±2.09	8.70±2.01	8.68±1.89		-0.03 (-1.42 to 1.36)	1.00	0.31 (-1.08 to 1.69)	1.00	0.27 (-1.07 to 1.61)	1.00
Week 8	8.93±1.36	9.67±2.01	9.42±1.28		-0.26 (-1.30 to 0.77)	1.00	0.60 (-0.49 to 1.69)	0.89	0.33 (-0.47 to 1.14)	1.00
**Pre-*β*-HDL, mg/L**										
Baseline	120.69±33.68	115.19±28.28	107.95±22.69	**<0.001**						
Week 4	112.65±21.18	117.95±32.88	107.74±38.90		-6.75 (-31.30 to 17.80)	1.00	7.92 (-11.04 to 26.88)	1.00	1.17 (-22.15 to 24.50)	1.00
Week 8	62.46±15.80	74.07±15.80	64.03±22.83		-6.59 (-23.51 to 10.34)	1.00	14.23 (-4.12 to 32.59)	0.244	7.65 (-11.13 to 26.42)	1.00
**Pre-β_1-HDL, mg/L**										
Baseline	69.12±22.20	67.51±17.56	64.09±15.29	**<0.001**						
Week 4	66.48±17.74	70.05±19.73	62.72±20.53		-5.66 (-20.15 to 8.83)	1.00	4.35 (-8.38 to 17.09)	1.00	-1.31 (-14.75 to 12.14)	1.00
Week 8	34.37±14.10	40.57±10.67	34.07±13.85		-4.83 (-14.63 to 4.98)	1.00	6.98 (-4.55 to 18.51)	0.661	2.15 (-9.22 to 13.52)	1.00
**Pre-β_2-HDL, mg/L**										
Baseline	51.57±19.13	47.68±14.79	43.86±11.28	**<0.001**						
Week 4	46.16±12.98	47.90±15.50	45.02±19.81		-1.13 (-13.89 to 11.63)	1.00	3.51 (-6.36 to 13.38)	1.00	2.38 (-10.30 to 15.06)	1.00
Week 8	28.09±12.81	33.51±9.22	29.96±13.16		-1.80 (-10.88 to 7.28)	1.00	7.19 (-2.69 to 17.08)	0.329	5.39 (-5.33 to 16.11)	1.00
**HDL_3, mg/L**										
Baseline	576.86±134.88	573.03±140.83	538.40±115.86	**<0.001**						

Table S9. Continued

Index	Cho (n = 23)	Cho-CT (n = 23)	Cho-MeCT (n = 23)	G×T Effect	Cho-MeCT vs.Cho-CT		Cho-CT vs.Cho		Cho-MeCT vs.Cho	
					MD (95% CI)	*P*	MD (95% CI)	*P*	MD (95% CI)	*P*
Week 4	530.96±154.58	546.50±111.66	513.20±91.77		-14.09 (-88.85 to 60.66)	1.00	17.67 (-76.05 to 111.39)	1.00	3.58 (-91.13 to 98.28)	1.00
Week 8	442.17±88.30	453.20±93.03	415.61±121.63		-18.38 (-102.21 to 65.44)	1.00	13.16 (-71.70 to 98.01)	1.00	-5.23 (-92.55 to 82.09)	1.00
**HDL_{3c}, mg/L**										
Baseline	81.79±22.16	85.29±24.62	84.30±19.06	0.958						
Week 4	75.25±19.34	78.84±24.43	80.68±16.57		2.43 (-11.91 to 16.77)	1.00	1.50 (-11.48 to 14.48)	1.00	3.94 (-9.11 to 16.99)	1.00
Week 8	86.05±18.40	90.18±24.96	93.58±19.81		3.99 (-12.57 to 20.55)	1.00	2.04 (-16.02 to 20.11)	1.00	6.04 (-8.76 to 20.84)	1.00
**HDL_{3b}, mg/L**										
Baseline	249.44±65.63	249.52±75.24	232.57±56.82	**<0.001**						
Week 4	247.20±94.97	251.56±80.35	221.32±62.46		-20.46 (-71.51 to 30.58)	1.00	4.31 (-59.19 to 67.80)	1.00	-16.16 (-78.11 to 45.79)	1.00
Week 8	157.46±52.60	172.74±81.91	148.84±72.68		-14.12 (-67.78 to 39.53)	1.00	15.23 (-38.35 to 68.80)	1.00	1.10 (-51.59 to 53.79)	1.00
**HDL_{3a}, mg/L**										
Baseline	245.63±92.34	238.21±70.55	221.53±62.95	**0.030**						
Week 4	208.51±56.66	216.11±69.13	211.20±51.29		3.26 (-43.88 to 50.40)	1.00	11.23 (-35.55 to 58.01)	1.00	14.49 (-27.88 to 56.96)	1.00
Week 8	198.66±34.20	190.28±48.43	173.19±39.14		-8.93 (-41.05 to 23.18)	1.00	-4.74 (-45.83 to 36.34)	1.00	-13.68 (-50.70 to 23.35)	1.00

Table S9. Continued

Index	Cho (n = 23)	Cho-CT (n = 23)	Cho-MeCT (n = 23)	G×T Effect	Cho-MeCT vs.Cho-CT		Cho-CT vs.Cho		Cho-MeCT vs.Cho	
					MD (95% CI)	*P*	MD (95% CI)	*P*	MD (95% CI)	*P*
**HDL_2, mg/L**										
Baseline	464.12±292.97	485.50±216.74	425.36±189.88	**<0.001**						
Week 4	453.31±157.96	443.39±170.93	457.24±108.41		40.11 (-59.94 to 140.16)	1.00	-19.26 (-113.18 to 74.66)	1.00	20.85 (-72.28 to 113.98)	1.00
Week 8	725.66±139.03	781.01±311.77	704.58±129.20		-50.17 (-245.34 to 144.99)	1.00	46.01 (-170.25 to 262.27)	1.00	-4.16 (-148.49 to 140.17)	1.00
**HDL_{2a}, mg/L**										
Baseline	264.38±81.64	265.84±76.13	242.46±80.89	**0.040**						
Week 4	278.51±82.42	271.38±71.82	256.48±57.67		-2.21 (-56.50 to 52.08)	1.00	-7.92 (-63.34 to 47.49)	1.00	-10.13 (-66.44 to 46.18)	1.00
Week 8	319.21±76.09	329.37±95.61	286.03±82.49		-30.65 (-88.49 to 27.19)	0.973	9.37 (-58.87 to 77.61)	1.00	-21.28 (-82.17 to 39.62)	1.00
**HDL_{2b}, mg/L**										
Baseline	199.74±242.01	219.65±179.57	182.91±150.71	**<0.001**						
Week 4	174.80±125.32	172.01±126.07	200.76±107.29		42.22 (-38.16 to 122.60)	0.995	-10.09 (-92.94 to 72.75)	1.00	32.12 (-51.53 to 115.77)	1.00
Week 8	406.46±93.68	451.64±237.04	418.55±96.12		-19.62 (-174.58 to 135.33)	1.00	37.89 (-125.69 to 201.46)	1.00	18.26 (-86.94 to 123.46)	1.00

Table S9. Continued

Index	Cho (n = 23)	Cho-CT (n = 23)	Cho-MeCT (n = 23)	G×T Effect	Cho-MeCT vs.Cho-CT		Cho-CT vs.Cho		Cho-MeCT vs.Cho	
					MD (95% CI)	*P*	MD (95% CI)	*P*	MD (95% CI)	*P*
**LCAT, nmol · L^{-1} · h^{-1}**										
Baseline	126.27±14.25	123.12±17.84	121.90±16.26	0.350						
Week 4	131.44±17.27	124.87±21.69	129.85±22.02		5.44 (-10.70 to 21.58)	1.00	-5.40 (-20.47 to 9.66)	1.00	0.04 (-15.48 to 15.55)	1.00
Week 8	141.75±17.82	129.02±12.38	136.57±16.82		8.00 (-4.28 to 20.29)	0.514	-11.55 (-24.14 to 1.04)	0.093	-3.55 (-17.50 to 10.40)	1.00
**CETP, nmol · L^{-1} · h^{-1}**										
Baseline	86.36±8.12	85.34±7.80	83.64±9.31	0.536						
Week 4	89.08±7.49	88.78±9.19	84.93±8.01		-3.14 (-10.22 to 3.94)	1.00	0.13 (-6.79 to 7.04)	1.00	-3.02 (-9.32 to 3.29)	1.00
Week 8	91.86±9.78	90.55±11.46	86.01±9.58		-3.83 (-11.93 to 4.28)	1.00	-0.89 (-9.09 to 7.32)	1.00	-4.71 (-11.63 to 2.21)	0.435

* Data are mean ± SD. Analyses were performed using generalized estimating equations (GEE). For the primary outcome (HDL-CEC), covariates included baseline measurement values, time, group, group-by-time interaction, and baseline body mass index (BMI). For the other outcomes, covariates included baseline measurement values, time, group, and group-by-time interaction (without baseline BMI adjustment). Pairwise comparisons were performed using linear contrasts with Bonferroni correction. Boldfaced P values denote significance after correction.

CETP, Cholesteryl ester transfer protein; Cho-CT, Brussels chicory supplementation combined with supervised concurrent resistance exercise and high-intensity interval training; Cho-MeCT, mindfulness-enhanced concurrent resistance exercise and high-intensity interval training with Brussels chicory; Con, control; HDL-C, high-density lipoprotein cholesterol; HDL-CEC, high-density lipoprotein cholesterol efflux capacity; LCAT, Lecithin: cholesterol acyltransferase; G×T, group-by-time interaction effect; MD, mean difference; CI, confidence interval.
